# A new promoter element associated with daily time keeping in *Drosophila*

**DOI:** 10.1093/nar/gkx268

**Published:** 2017-04-12

**Authors:** Brandi Sharp, Eric Paquet, Felix Naef, Akanksha Bafna, Herman Wijnen

**Affiliations:** 1 Department of Biology, University of Virginia, Charlottesville, VA 22903, USA; 2 Institute of Bioengineering, School of Life Sciences, Ecole Polytechnique Fédérale de Lausanne, Lausanne, Switzerland; 3 Biological Sciences and Institute for Life Sciences, University of Southampton, Southampton SO17 1BJ, UK

## Abstract

Circadian clocks are autonomous daily timekeeping mechanisms that allow organisms to adapt to environmental rhythms as well as temporally organize biological functions. Clock-controlled timekeeping involves extensive regulation of rhythmic gene expression. To date, relatively few clock-associated promoter elements have been identified and characterized. In an unbiased search of core clock gene promoters from 12 species of *Drosophila*, we discovered a 29-bp consensus sequence that we designated as the Clock-Associated Transcriptional Activation Cassette or ‘CATAC’. To experimentally address the spatiotemporal expression information associated with this element, we generated constructs with four separate native CATAC elements upstream of a basal promoter driving expression of either the yeast *Gal4* or firefly l*uciferase* reporter genes. Reporter assays showed that presence of wild-type, but not mutated CATAC elements, imparted increased expression levels as well as rhythmic regulation. Part of the CATAC consensus sequence resembles the E-box binding site for the core circadian transcription factor CLOCK/CYCLE (CLK/CYC), and CATAC-mediated expression rhythms are lost in the presence of null mutations in either *cyc* or the gene encoding the CLK/CYC inhibitor, *period* (*per*). Nevertheless, our results indicate that CATAC's enhancer function persists in the absence of CLK/CYC. Thus, CATAC represents a novel *cis*-regulatory element encoding clock-controlled regulation.

## INTRODUCTION

The circadian clock of higher eukaryotes is understood to be a conserved transcription/translation auto-regulatory feedback mechanism controlled via rhythmic transcriptional activation and repression. Mammals as well as insects have circadian clocks that operate via interlocking transcriptional feedback loops that rely on heterodimers of basic helix–loop–helix (bHLH), Per-Arnt-Sim (PAS) domain transcription factors (TFs) for transcriptional activation (1). In the clock circuit of the fruit fly, *Drosophila melanogaster*, a heterodimer consisting of CLOCK (CLK) and CYCLE (CYC), acts as the core transcription factor (2,3). CLK/CYC binds DNA by associating with a canonical CACGTG E-box or E-box-like sequences (4–6). The detection of transcripts regulated by CLK/CYC is of particular interest to the field of circadian biology. E-boxes, however, are poor predictors of potential clock-regulated genes because the sequence motif is widespread throughout the fly genome. The nucleotides flanking the E-box and/or an arrangement of closely spaced associated motifs, on the other hand, likely contribute to clock transcriptional activity, TF specificity and increased binding affinity (7,8).

In *Drosophila*, studies of the promoter regions of known clock genes, *period (per*) and *timeless* (*tim*), found that E-box-dependent enhancers are necessary for circadian transcriptional modulation. The *per* promoter has, arguably, the best studied circadian enhancer motif to date. The enhancer is a 69-bp sequence upstream of the transcription start site (TSS) that activates circadian gene expression (5). This enhancer relies on an E-box motif to mediate transcriptional activation and 3΄ sequences near the E-box to drive strong amplitudes and tissue specific expression (5,9). However, the core of the 69-bp enhancer is actually an E-box-bearing 18-bp region that is able, when multimerized, to recapitulate *per* spatial and temporal expression (4). The *tim* promoter possesses closely spaced E- and TER (*tim*E-box-like repeats) boxes, which are a variant of the consensus E-box sequence (6). This *tim* enhancer relies on two non-canonical E-boxes, TER1 and TER2, to strongly initiate gene expression and cycling amplitude. The canonical E-box present in the enhancer appears to be non-functional on its own and seems to require the TER boxes to elicit functionality.

Increasing knowledge of the *Drosophila* genome, annotated and cloned full-length cDNAs, transcription start sites (TSSs) and transcription factor binding sites (TFBSs) have provided more tools to determine the elements responsible for transcriptional regulation (10,11). In previous work (12), we identified a conserved motif consisting of two closely spaced E-box-like elements in the 69-bp *per* enhancer as well as in other CLK/CYC-controlled genes (*tim, vrille, Par domain protein 1* and *clockwork orange*). As a continuation of this work, we, hereby, identified a second novel and independent 29-bp motif. The present study describes spatiotemporal expression information contributed by the latter motif, which was named Clock-Associated Transcriptional Activation Cassette or ‘CATAC’.

## MATERIALS AND METHODS

### Identification of CATAC consensus sequence

**Figure 1.**
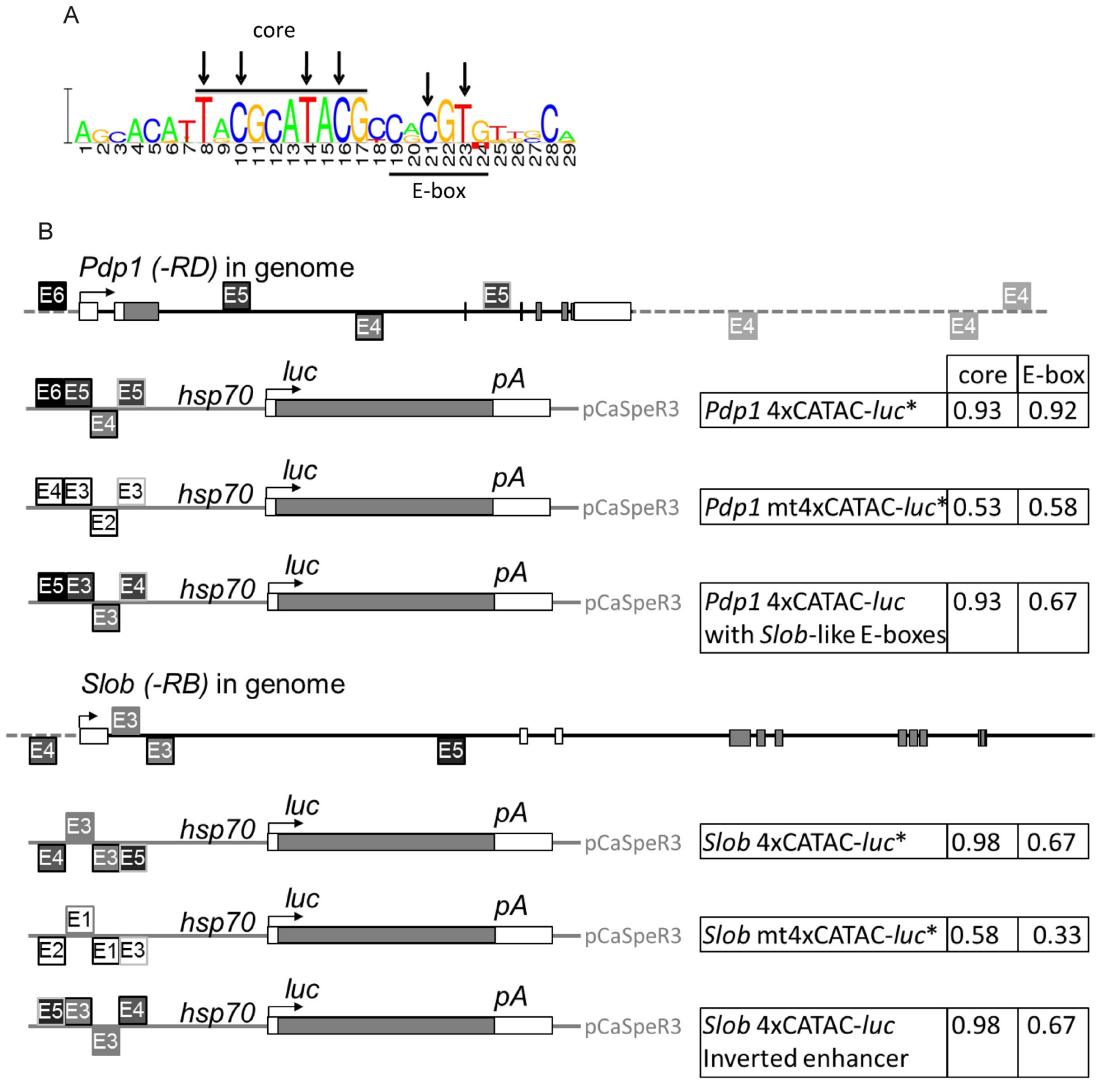
Clock-associated transcriptional activation cassette (CATAC) and reporter constructs. (**A**) Consensus sequence for the CATAC element derived from five clock genes (*tim, per, cwo, vri* and *Pdp1*) from 12 *Drosophila* species. The highly conserved core region is overlined and the E-box sequence match is underlined. Residues mutated in this study are marked with arrows. (**B**) CATAC-*luciferase* reporter constructs used in this research. Reporters were generated by inserting CATAC sequences from the *Pdp1* or *Slob* genes upstream of the basal promoter in the pPT*luc* vector. Individual CATAC elements are indicated as E1/E2/E3/E4/E5/E6 depending on the match of their E-box region to the CACGTG consensus (1–6 matching residues). The native CATAC elements used for the *Pdp1* and *Slob* CATAC constructs are also shown in their genomic context with the *Pdp1-RD* and *Slob-RB* transcripts, respectively (the patterns of shading and outline color allow matching between transgenic elements and their genomic origin). The mt4xCATAC constructs contain six (A ⇔ T or C ⇔ G) transversion mutations in each of the CATAC elements at the positions indicated in (A). ‘Pdp1 4xCATAC-*luc* with *Slob*-like E-boxes’ (PSE) was created by changing the E-box consensus matches of the original elements as indicated. The ‘core’ and ‘E-box’ scores for each construct represent the fraction of residues in each of the four elements matching the respective TRCGCATACG and CRCGTG consensus sequences from (A). For constructs marked with an asterisk, additional versions carrying *Gal4* instead of *luc* as the reporter were generated. Sequences are specified in Materials and Methods.

The bioinformatics model used to predict the ‘CATAC’ element is identical to that previously described for the discovery of the E1–E2 element (12). Briefly, we used MEME (13) to scan the genomic sequences in windows of 2.5 kb around the TSSs of *tim, vrille, Pdp1* and *cwo (stich1)* in 12 *Drosophila* species (MultiZ alignments were downloaded from UCSC, dm3 assembly). This identified a conserved set of CATAC sequences (Supplementary Figure S1). In order to obtain our final position weight matrix (Figure 1A), we trained a Hidden Markov Model as in (12), but consisting only of a single 29 bp motif, using the MEME-derived consensus as a seed. A .bed file that identifies all CATAC sites with a bit score >5 in the *Drosophila melanogaster* genome (BDGP R5/dm3) is included in the supplemental information.

### Generating CATAC reporter constructs

Synthetic oligonucleotides were designed to match four separate, native occurrences of the CATAC element from either the *Pdp1* or *Slob* promoters: *Slob* 4xCATAC enhancer, 5΄pGGCCGATAACGCGGCGTATGCGCAATGTCGAAGCATATTACGCATACGCCCCATCCGC; 5΄pGTTTGCTGTGGCGGATGGGGCGTATGCGTAATATGCTTCGACATTGCGCATACGCCGCGTTATC; 5΄pCACAGCAAACGCTGCGTATGCGTAATACTTTGTGCACACGTTGCGTATGAGTAATGTCCT; 5΄pAATTAGGACATTACTCATACGCAACGTGTGCACAAAGTATTACGCATACGCAGC, *Slob* mt4xCATAC enhancer, 5΄pGGCCGATATCCCGGCCTTTGCCCTATGTCGAAGCATATAAGGCAAGGCCCGAACCGC; 5΄pGATTGCTGTGGCGGTTCGGGCCTTTGCCTTATATGCTTCGACATAGGGCAAAGGCCGGGATATC; 5΄pCACAGCAATCCCTGCCTTTGCCT TATACTTTGTGCACTCCTTGCCTTTGACTTATGTCCT; 5΄pAATTAGGACATAAGTCAAAGGCAAGGAGTGCACAAAGTATAAGGCAAAGGCAGG, *Pdp1* 4xCATAC enhancer, 5΄pGGCCAGCACATTACGCATACGTCACGTGTTGCAAAAATAATACGTATACGACGCGTGTC; 5΄pTGGTACAGAAGACACGCGTCGTATACGTATTATTTTTGCAACACGTGACGTATGCGTAATGTGCT; 5΄pTTCTGTACCATGCGGCGTATGAGCAATCTGTTAATACGTTACCCATACGCCCCGTGGGC C; 5΄pAATTGGCCCACGGGGCGTATGGGTAACGTATTAACAGATTGCTCATACGCCGCA, *Pdp1* mt4xCATAC enhancer, 5΄pGGCCAGCACATAAGGCAAAGGTCAGGAGTTGCAAAAATAAAAGGTAAAGGACGGGAGTC; 5΄pAGGTACAGAAGACTCCCGTCCTTTACCTTTTATTTTTGCAACTCCTGACCTTTGCCTTATGTGCT; 5΄pTTCTGTACCTTC CGGCCTTTGACCTATCTGTTAATACGTA AGCCAAAGGCCCGGAGGGCC; 5΄pA ATTGGCCCTCCGGGCCTTTGGCTTACGTATTAACAGATAGGTCAAAGGCCGGA, *Pdp1* 4xCATAC with *Slob*-like E-boxes enhancer, 5΄pGGCCAGCACATTACGCATACGTAACGTGTTGCAAAAATAATACGTATACGAAGCGTTTC; 5΄pTCGTACAGA AGAAACGCTTCGTATACGTATTATTTTTGCAACACGTTACGTATGCGTAATGT GCT; 5΄pTTCTGTACGATGGGGCGTATGAGCAATCTGTTAATACGTTACCCATACGCCGCGTTGGC C; 5΄pAATTGGCCAACGCGGCGTATGGGTAACGTATTAACAG ATTGCTCATACGCCCCA. Oligos were annealed to match their order and orientation in the native *Pdp1* or *Slob* promoters. Flanking EagI and EcoRI sequences allowed for the 4xCATAC enhancers to be inserted upstream of the *hsp70* basal promoter in the pPTGAL vector (14) (obtained from the Drosophila Genomics Resource Center). The luciferase constructs (pPT*luc*) were created by blunt-end cloning to replace a 3193 bp fragment containing *Gal4* flanked by *Pst*I sites with a 1955 bp HindIII–BamHI fragment containing the *luciferase* gene from pGL3-Basic (Promega) (15). Copies of pPT*luc* can be requested online (http://www.addgene.org/Herman_Wijnen/). For each reporter construct, several independent lines were generated.

### Drosophila stocks

Fly stocks bearing the *w^1118^*, *UAS-CD8::GFP*, and *eya^2^* mutations were obtained from the Bloomington Drosophila Stock Center. The *cyc^01^, per^01^* and *tim-luc* alleles have been described previously (3,16,17). All stocks were maintained on standard yeast cornmeal agar food.

### *In vivo* luciferase monitoring

Bioluminescence monitoring of flies was carried out as described previously (17–19). 100 μl of a 5% sucrose 1% agar solution containing 15 mM luciferin (GOLDBIO) was added to every other well of a white 96-well microtiter plate (Optiplate, Perkin Elmer). Flies were entrained for 3 days in a 12-h light, 12-h dark cycle (LD 12:12). On day 3 during the light phase, flies were anesthetized, added to separate wells and covered with clear plastic domes to reduce noise caused by fly movement closer to and farther away from the photodetector. Plates were then placed into a TopCount Scintillation Counter and subjected to the remaining portion of the LD phase and subsequent constant darkness (DD). Luminescence from each fly was monitored for 7–17 s per time point, thereby allowing data for each fly to be collected roughly once every hour.

### Quantitative data analysis

Luciferase assay data were analyzed for period in the circadian range (15–35 h) and relative amplitude error (RAE) by an iterative, coupled fast Fourier transform-non-linear least squares (FFT-NLLS) multicomponent cosine analysis (19). RAE is the ratio of the 95% confidence interval by the amplitude estimate—the ratio of amplitude error to most probable amplitude. Lower RAE values suggest stronger rhythms. In our analysis, individual flies with RAE values <0.7 were considered rhythmic, ≥0.7 were considered weakly rhythmic, and those for which the program returned no data (RAE > 1), arrhythmic. The first 24-h of data were excluded from the plots and quantitative analysis since flies placed on fresh luciferin media require time to inactivate previously synthesized luciferase. For each fly, only DD data were analyzed for statistical analysis.

### Imaging of GFP and luciferase reporters

Larvae and adult flies producing 4xCATAC-*Gal4* driven expression of membrane-tethered green fluorescent protein were dissected in Ringer's solution (20) under a dissecting scope equipped with UV light. Tissues expressing GFP were wet or dry mounted and immediately imaged with a fluorescence microscope equipped with a CCD camera. Luciferase expressing flies were dehydrated for 24-h then fed on a cotton plug soaked in 200 μl of a 1% sucrose solution containing 15 mM luciferin (GOLDBIO). Feeding occurred for 1–2 h prior to imaging of male heads and for 25–26 h prior to imaging of male bodies. Luciferase-expressing adult fly heads or bodies were mounted on a sterile filter insert (Millicell-CM; Millipore Inc.) and immobilized under a 13-mm coverglass with the help of sterile vacuum grease (21). The insert was then placed in a sterile glass-bottom dish (FluoroDish FD35PDL) containing insect tissue culture media with 0.1 mM luciferin. Sterile vacuum grease was then applied to seal the dish. Luminescence imaging was conducted as previously described by Sellix *et al.* (21), with an inverted epifluorescence microscope (Olympus CKX-41 equipped with a cooled intensified CCD camera (Mega10Z; Stanford Photonics Inc., Palo Alto, CA, USA) housed in a light-tight wooden dark box. Luminescence images were collected using Piper image analysis software (Stanford Photonics).

### Quantitative reverse transcriptase PCR (qRT-PCR) analyses

qRT-PCR expression analyses were carried out as described previously (22,23). *Slob* or *Pdp1* 4xCATAC-*luciferase* flies were entrained to 12 h light/12 h dark cycles at 25°C prior to release into constant conditions (DD 25°C). Flies were harvested onto dry ice at time points CT0, CT6, CT12, CT18 and CT24, and adult heads were dissected on a chilled platform and transferred to guanidinium thiocyanate buffer. Four separate groups of flies (∼50 each) were used for each experimental condition. Total RNA was obtained from the heads using the RNAqueous4PCR kit (Ambion). Sample concentration and purity was analysed using a NanoDrop spectrophotometer. Samples exhibited OD 260/280 ratios between 1.8 and 2.1. Concentrations were adjusted to 25 ng/μl in 10 mM Tris–HCl 0.1 mM EDTA pH 8.0 buffer and samples were frozen at –80°C in aliquots until further use. The RNA samples were then analyzed with the SensiFAST SYBR No-ROX One-Step qPCR Kit (Bioline) using experimental primer pairs designed to specifically amplify fragments of the circadian *Pdp1 or Slob* transcripts, the transgenic *luciferase* transcript or the *EF1β* control transcript (see Supplementary Table S1). All primers and amplicons used in this study have been described before (22,23). Oligonucleotides were sourced from Integrated DNA Technologies (25 nmol scale, standard desalting purification). No-template and RNAse-treated controls were included to avoid false positive results. The following thermocycling protocol was used for the *Pdp1* and *Slob* amplicons: 60°C 180 s, 95°C 300 s, [95°C 15 s, 62°C 30 s] ×45, 40°C 60 s, melting curve 60°C to 95°C 0.2°C/s. Thermocycling for *luciferase* and *EF1β* made use of an adjusted annealing temperature of 60°C instead of 62°C during the 45 amplification phase. Expression levels measured on a SmartCycler system (Cepheid) relative to *EF1β* were determined using the comparative Cycle threshold (Ct) method (24) and analysis of timed gene expression was carried out after normalization to the time course average. Amplicon sizes and specificity were verified by 2% agarose gel electrophoresis of all experimental and control PCR samples. Statistical analyses were conducted using SPSS.

## RESULTS

### Identification of a conserved promoter element in *Drosophila*

In order to detect over-represented *cis*-acting elements associated with clock gene expression, we combined MEME (13) with a Hidden Markov Model ((12) and Materials and Methods) to analyse an alignment of promoter sequences of the core clock genes (*per, tim, Pdp1, cwo* and *vri*) for 12 species of *Drosophila*. As a result, a 29-bp motif designated the Clock-Associated Transcriptional Activation Cassette or ‘CATAC,’ was identified as over-represented in these promoters (Figure 1A, Supplementary Figure S1). The consensus bears a well-conserved core' motif (nucleotides 8–17) and, although the model was not seeded with an E-box, it bears an E-box-like motif as well (nucleotides 19–24) (Figure 1A). To our knowledge, no functional analyses of individual CATAC elements or corresponding genomic sites have been reported to date. An illustration of the relationship between the CATAC element and the major known circadian enhancer element in the *per* gene (69-bp enhancer (5)) is provided in supplemental Supplementary Figure S1B.

A possible association of CATAC with clock-controlled and/or circadian transcription was explored further by determining the relative frequencies at which the CATAC element occurred in relevant promoter sequences outside of the five training genes. In particular, enrichment of CATAC was found in the top 0.5% genes (n = 61 genes) predicted to be induced upon activation of the circadian regulator CLK (25) as well as in the top 0.7% genes (*n* = 97 genes) encoding transcripts predicted to exhibit circadian oscillations (25–28) (Supplementary Figure S2). A prominent circadian transcript associated with CATAC promoter elements is *Slob* (*Slowpoke binding protein*) (27). With the exception of the element in the first intron of *per*, all CATAC elements associated with these six genes coincide with conserved sequence elements predicted by PhastCons analysis from an alignment of 27 insect genomes (29).

Based upon the sequence alignment, conservation, and subsequent CLK/CYC target gene enrichment analyses, CATAC was hypothesized to be a regulatory element involved in mediating spatiotemporal expression of clock-regulated genes.

### CATAC imparts rhythmicity to a luciferase reporter

To test our hypothesis, we studied CATAC elements found in two genes, *Pdp1* and *Slob*, that not only exhibit strong CLK/CYC-associated circadian oscillations, but also have an unusually high number of CATAC motifs. The *Pdp1* gene encodes a clock component (PDP1-ε) that impacts molecular circadian oscillations, particularly in the clock neurons, as well as circadian behaviour (30,31). Remarkably, *Pdp1* has seven CATAC elements in close proximity, one of which bears a canonical E-box (Figure 1B). The *Slob* gene, which encodes proteins that regulate the slowpoke channel, exhibits strong circadian regulation of its expression. The *Slob* promoter has four CATAC elements which all possess noncanonical E-boxes. To address the spatiotemporal expression information associated with CATAC, enhancer elements were generated, bearing four separate native CATAC elements from the *Slob* or *Pdp1* promoters (Figure 1B; Supplementary Figure S3). Each enhancer was assembled such that the four CATAC elements matched their order and orientation in the native *Pdp1* and *Slob* promoters (Figure 1B; Supplementary Figure S3). Moreover, like their promoters, the *Pdp1* 4xCATAC enhancer possesses one CATAC element with a canonical E-box while the *Slob* 4xCATAC enhancer has four noncanonical E-box-bearing CATAC elements. More specifically, in comparison with the *Pdp1* 4xCATAC enhancer the *Slob* 4xCATAC enhancer has more mismatches relative to both the CACGTG canonical E-box consensus (9/24 versus 4/24 mismatches) as well as the CRCGTG consensus for the E-box-like element within CATAC (Figure 1A; 8/24 versus 2/24 mismatches).

**Figure 2.**
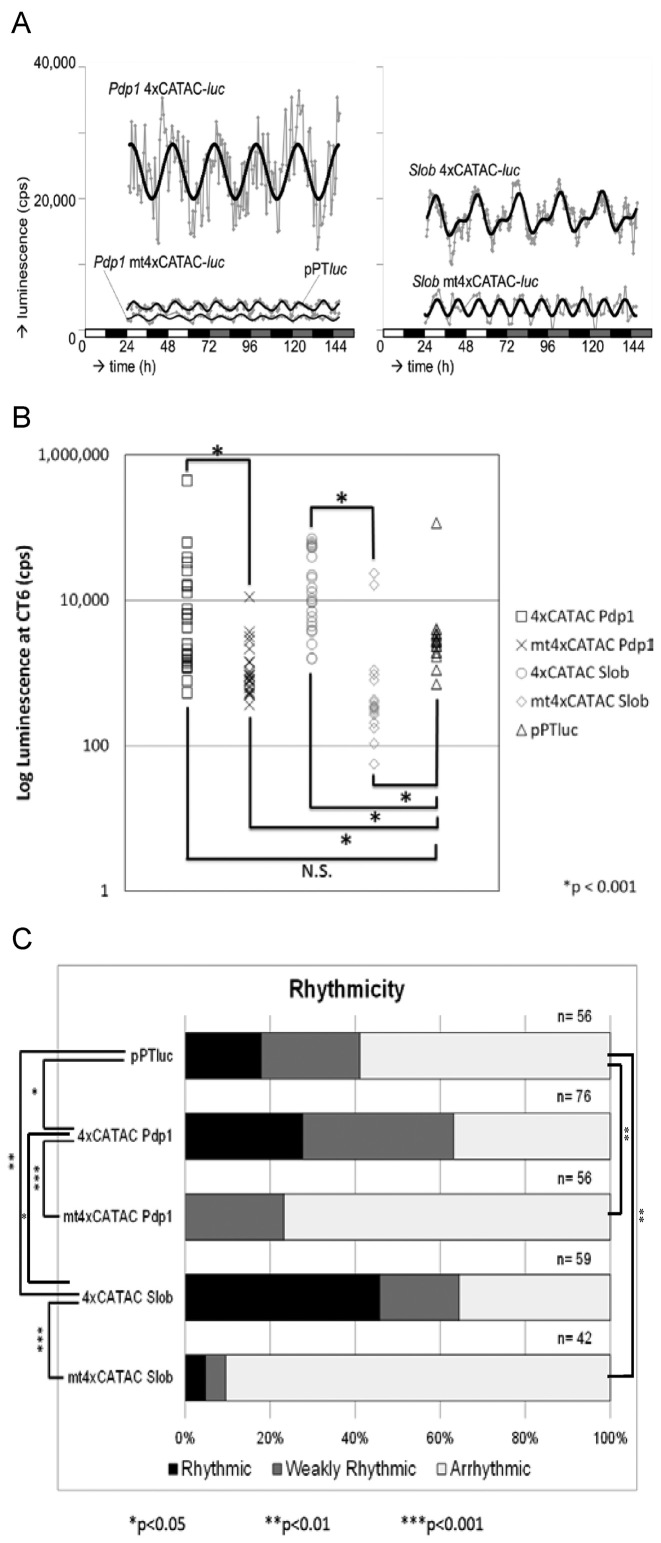
Regulation of 4xCATAC-*luc* reporter constructs. (**A**) Comparison of detrended, average traces (gray) and their corresponding FFT theoretical overlays (black) demonstrated a strong rhythm in wild-type CATAC*-luc* reporter activity. Average traces were derived from 26 *Pdp1* 4xCATAC*-luc*, 23 *Pdp1* mt4xCATAC*-luc* and 19 pPT*luc* lines (left panel) as well as 23 *Slob* 4xCATAC*-luc* and 17 *Slob* mt4xCATAC*-luc* lines (right panel). The luminescence profiles of reporters with mutant CATAC elements showed weaker, ultradian rhythms that resembled those observed for the enhancer-less control (pPT*luc*). (**B**) 4xCATAC-induced reporter expression was determined after 6 h in DD from non-detrended, averaged raw data. The expression levels of *Pdp1* and *Slob* 4xCATAC were higher than their mutant counterparts (*P* < 0.001, Mann–Whitney U/Wilcoxon W rank sum test). The pPT*luc* expression levels were significantly higher than *Pdp1* and *Slob* mt4xCATAC but lower than *Slob* 4xCATAC. (**C**) 4xCATAC*-luc* reporter rhythmicity was determined from TopCount *in vivo* luminescence data by the FFT-NLLS utility of the BRASS software program. Individual flies with relative amplitude error (RAE) <0.7, between 0.7 and 1, or >1 were classified as rhythmic, weakly rhythmic and arrhythmic, respectively. Pairwise Chi-square test comparisons were used to generate the indicated *P* value estimates. *Pdp1* and *Slob* 4xCATAC generated greater rhythmicity than their mutated counterparts. Residual rhythmicity was observed for empty pPT*luc* vector, and to a lesser degree in mutant 4xCATAC constructs, but this was always significantly reduced relative to the wild-type *Pdp1* and *Slob* 4xCATAC reporters.

Mutated CATAC constructs were also generated by making transversion mutations to each of the four native elements. Mutations were made to the well-conserved core motif at residues 8, 10, 14 and 16, as well as to the E-box-like motif at residues 21 and 23 (Figure 1A; Supplementary Figure S3). Native and mutated 4xCATAC enhancers were then inserted upstream of a basal promoter driving expression of the firefly *luciferase* reporter gene (Figure 1B). Multiple independent transgenic lines were generated by P element transformation of native and mutated 4xCATAC *Pdp1* or *Slob* reporter constructs. Transformants were tested in an automated bioluminescence assay (TopCount) which allowed measurement of the transcriptional reporter constructs at relatively high frequency in vivo. Native *Pdp1* and *Slob* 4xCATAC both showed strong luciferase expression (Figure 2, Supplementary Figure S4) and rhythms (Figure 2, Supplementary Figure S5; Table 1), and their average profiles showed a single peak per 24 h (Figure 2). Thus, the presence of a canonical E-box, which was lacking from the *Slob* 4xCATAC reporter, did not seem to be required for either rhythms or induction. A similar conclusion was reached in a previous study of the *tim* promoter, which showed that noncanonical E-boxes can significantly contribute to circadian transcriptional activity (6). If anything, native *Pdp1* 4xCATAC transgenes, which included better matches to both the canonical and CATAC E-box consensus sequences tended to exhibit somewhat lower levels of rhythmicity than native *Slob* 4xCATAC transgenes with poorer quality E-boxes (Figure 2, Supplementary Figure S5; Table 1).

**Table 1.** Comparison of in vivo luminescence rhythms of different 4xCATAC*-luc* reporters

Genotype	#lines	#flies	%R	%WR	%AR	Mean RAE ± SEM	Mean period ± SEM
*Pdp1* 4xCATAC*-luc*	26	76	28	36	37	0.69 ± 0.02^a,b^	24.09 ± 0.26^g^
*Pdp1* with *Slob*-like E-boxes 4xCATAC*-luc*	18	105	44	25	31	0.62 ± 0.02^c^	23.01 ± 0.30
*Pdp1* mt4xCATAC*-luc*	23	56	0	23	77	0.90 ± 0.02^a,c,d,e^	20.29 ± 0.80^g,h^
*Slob* 4xCATAC*-luc*	23	59	46	19	36	0.57 ± 0.02^b,f^	24.72 ± 0.53^h^
Flipped *Slob* 4xCATAC*-luc*	8	71	32	31	37	0.68 ± 0.03^d^	23.17 ± 0.53
*Slob* mt4xCATAC*-luc*	17	42	5	5	90	0.71 ± 0.07	24.15 ± 0.83
pPT*luc*	19	56	18	23	59	0.71 ± 0.04^e,f^	23.29 ± 0.71

RAE: Relative Amplitude Error, only provided for weakly rhythmic and rhythmic flies. %R/WR/AR: percentage of single fly luminescence traces that are rhythmic, weakly rhythmic or arrhythmic.

ANOVA with post-hoc Tamhane's T2 *P* < 0.001^a,d,e^; *P* < 0.01^b,c,g,h^; *P* < 0.05^f^.

When we mutated all elements in the *Pdp1* and *Slob* 4xCATAC enhancers, the luciferase expression levels decreased (Figure 2, Supplementary Figure S4) and the rhythms were disrupted (Figure 2, Supplementary Figure S5; Table 1). Thus, the CATAC consensus sequence contributes to reporter induction and rhythmicity. Empty vector (pPT*luc*) and both mt4xCATAC constructs shared characteristically low level expression (Figure 2, Supplementary Figure S4) and comparable oscillation patterns, with two-peaks occurring per 24-hrs (Figure 2, Supplementary Figure S6). Earlier studies also found this 12-hr rhythm component in mutant E-box and basal promoter controls (4) and it has been reported to be a component of all expression patterns in such bioluminescence assays (4,19,32).

Although pPT*luc* and both of the mt4xCATAC constructs exhibited residual rhythmicity, neither exhibited the proportion of rhythmic flies observed for native *Pdp1* or *Slob* 4xCATAC (Figure 2). The residual rhythm for these constructs may have arrisen due to cryptic regulatory element(s) present in the reporter vector or, alternatively, as a result of rhythmic post-transcriptional modulation of luciferase reporter activity in the tissues expressing these constructs. We also generated two additional constructs, ‘flipped *Slob’* (FS) in which the orientation of *Slob* 4xCATAC in the vector was inverted and ‘*Pdp1* 4xCATAC with *Slob*-like E-boxes’ (PSE) in which the four E-boxes of *Pdp1* 4xCATAC were mutated such that their sequence fidelity matched that of the four E-boxes in *Slob* 4xCATAC (Figure 1B; Supplementary Figure S3). These constructs also exhibited better rhythmicity than pPT*luc* and both mt4xCATACs (Supplementary Figure S5; Table 1). Moreover, FS and PSE induced significantly more luciferase activity than the respective *Slob* and *Pdp1* mt4xCATAC controls (Supplementary Figure S4). Thus, in sum, in vivo luciferase reporter analyses uncovered preferential activity induction and rhythmicity for constructs containing wild-type CATAC elements.

We performed ANOVA analyses of luminescence data using only rhythmic and weakly rhythmic individual flies as determined by FFT-NLLS. The coherence of rhythms observed for *Pdp1* and *Slob* 4xCATAC reporters, as measured by the inversely correlated Relative Amplitude Error (RAE), was significantly stronger than those for mt4xCATAC and empty vector controls, respectively (Table 1). Period measurements, did not differ significantly between reporter constructs, except for the *Pdp1* 4xCATAC versus *Pdp1* mt4xCATAC comparison (Table 1). However, the unexpected short period length observed for *Pdp1* mt4xCATAC rhythms may be attributable to reduced accuracy in period estimation due to weak rhythms for this reporter.

### CATAC-driven reporters are expressed in the eye and other tissues with peak transcript phases during (subjective) day

**Figure 3.**
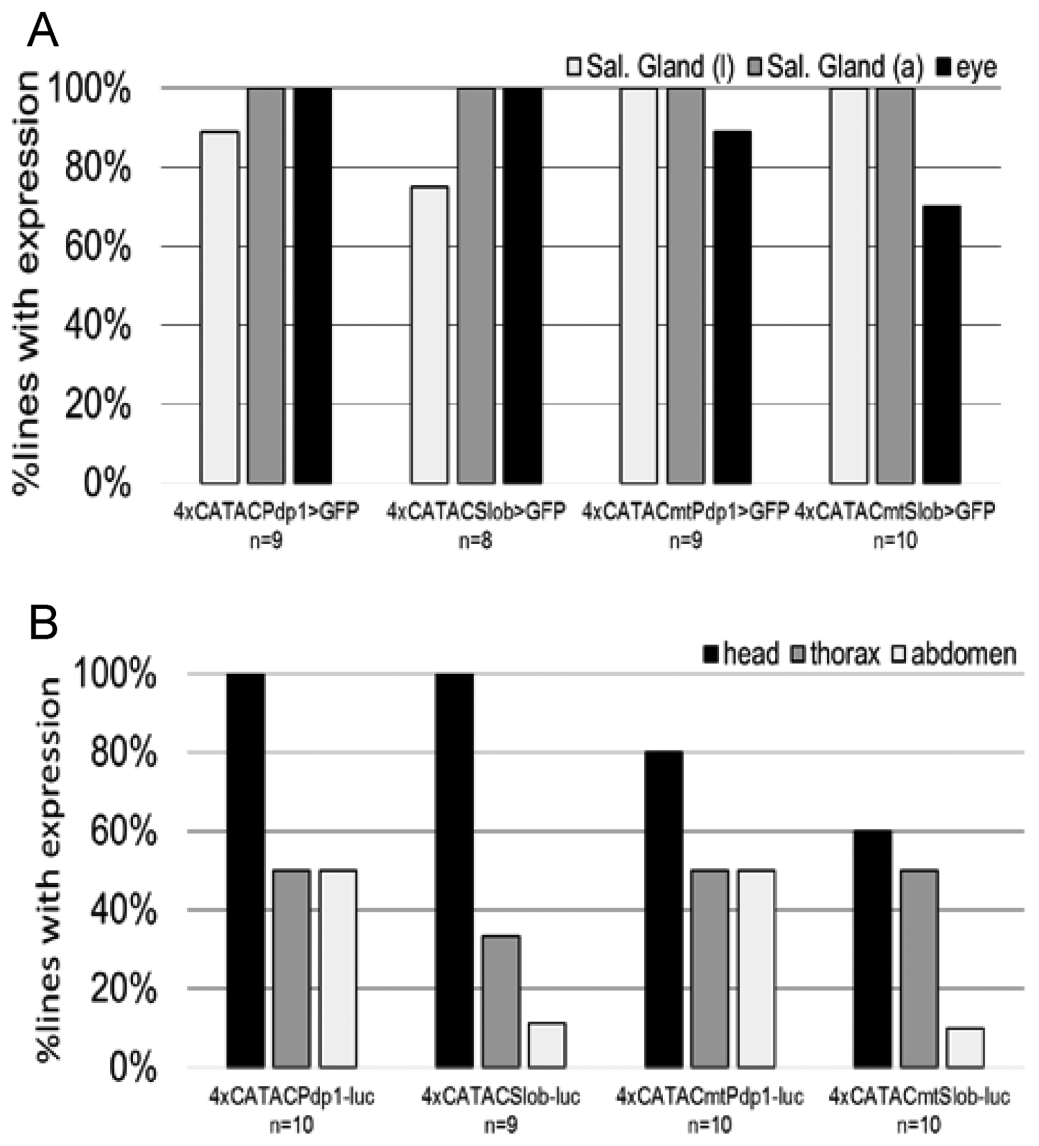
Spatial expression patterns of transgenic CATAC reporters. Larvae (l) and adult (a) male flies from multiple (n) lines expressing either 4xCATAC-*Gal4*-mediated GFP (**A**) or 4xCATAC-*luciferase* (**B**) were dissected to determine the spatial expression pattern of CATAC by fluorescence or luminescence imaging, respectively. The proportion of lines with tissues positive for a given reporter is indicated.

To further examine the spatiotemporal expression of the CATAC reporter, we crossed our 4xCATAC-*Gal4* lines with *UAS-CD8::GFP* flies to generate offspring that report CATAC activity by expression of membrane-tethered green fluorescent protein. In larvae and adult flies, 4xCATAC expression primarily included the salivary glands in addition to the photoreceptor cells of the compound eye in the adult (Figure 3A, Supplementary Figure S7). We also examined brains of the 4xCATAC flies but found no evidence of GFP expression. Little is known about the salivary glands as a circadianly rhythmic tissue and, although the observed signal in salivary glands was not due to autofluorescence or leaky expression of the *UAS-CD8::GFP* transgene (Supplementary Figure S7B), it is possible that the reporter gene expression in this tissue reflects the presence of a suspected salivary gland enhancer in the *hsp70* sequences (33) included in the pPTGal vector. Conversely, the photoreceptors are known to possess an autonomous, circadian oscillator (34). Furthermore, both the native *Slob* and *Pdp1* transcripts are expressed in the photoreceptors (30,35).

To follow-up, we imaged dissected heads and bodies of male *Pdp1* and *Slob* 4xCATAC luciferase flies that were fed a sucrose solution containing 15mM of luciferin, as in the TopCount assays. These tissues were then cultured in luciferin-containing media and imaged as close to their LD peak expression time as possible. The peak times were determined from the previously described TopCount assays (Figure 2) – *Pdp1* (ZT 2–3) and *Slob* (ZT 3–5). Heads showed luciferase expression in not only the compound eyes, but also the proboscis and antennae; each of which has been shown to possess a circadian oscillator (17,18) (Figure 3B, Supplementary Figure S8A). The bodies (including appendages) demonstrated signal in the wing, another tissue with an oscillator, and in other tissues within the thorax and abdomen (Figure 3B, Supplementary Figure S8B).

Given the status of the adult compound eye as a tissue with not only native expression of *Pdp1* and *Slob* (30,35) but also >80% of the circadian oscillators in fly heads (36), follow-up experiments were conducted to determine the contribution of the compound eyes to overall 4xCATAC reporter signal. We genetically removed the eyes via the eyes absent mutation, *eya^2^* (37). While the luciferase expression levels in these flies did decrease, there was still significant luciferase activity (Supplementary Figure S9, Table 2). Thus, while the eyes are a source of 4xCATAC driven luciferase signal, they are not its only relevant source.

**Table 2.** Impact of the eyeless mutant *eya^2^* on 4xCATAC*-luc* in vivo luminescence rhythms

genotype	#flies	%R	%WR	%AR	mean RAE±SEM	mean period±SEM
*eya^2^/CyO; Pdp1 4xCATAC-luc*	18	44	33	22	0.67±0.03	23.03±0.68
*eya^2^; Pdp1 4xCATAC-luc*	19	16	26	58	0.68±0.06	24.71±2.28
*eya^2^/CyO; Slob 4xCATAC-luc*	21	38	33	29	0.71±0.03	25.03±1.09
*eya^2^; Slob 4xCATAC-luc*	9	44	44	11	0.67±0.05	22.15±1.17

Therefore, spatial expression analyses of the wild-type and mutant 4xCATAC reporters uncovered expression in a number of tissues including the known oscillators of the compound eye. However, despite some trends suggesting a role for eye clocks in CATAC-mediated rhythmicity, no statistically significant differences between the spatial expression patterns of wild-type and mutant 4xCATAC reporters were uncovered (Figure 3, Supplementary Figure S8). As described above, this stands in contrast to clear differences in rhythmicities (Figure 2, Supplementary Figure S5, Table 1) and expression levels (Figure 2, Supplementary Figure S4, Table 1) produced by wild-type versus mutant 4xCATAC-*luc* constructs. Taken together, these findings suggest that CATAC primarily specifies temporal rather than spatial information.

**Figure 4.**
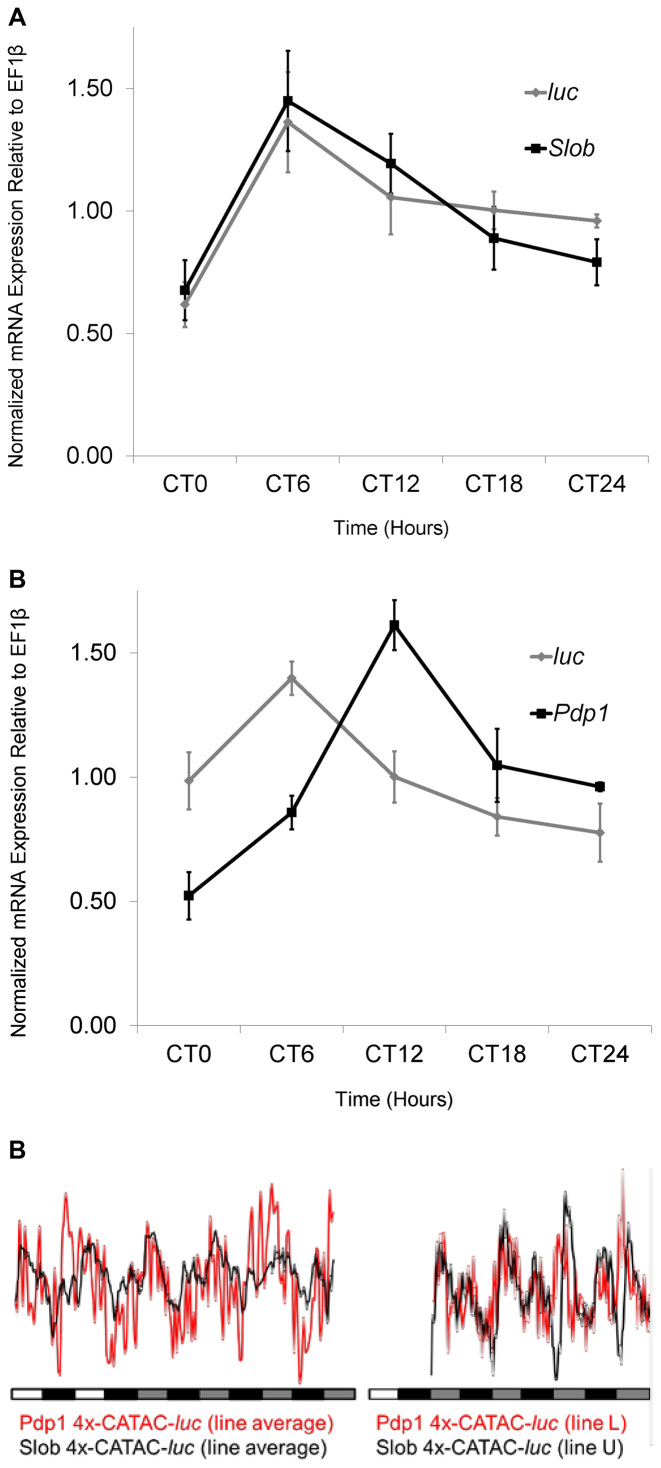
Time course of mRNA expression of *Pdp1* and *Slob* 4xCATAC-*luc* relative to their respective native gene transcripts in fly heads. (A, B)Transcript profiles were determined relative to *EF1β* by qRT-PCR for (**A**) *Slob* 4xCATAC*-luc* (Line U) and (**B**) *Pdp1* 4xCATAC*-luc* (Line L) homozygotes (gray diamonds with gray lines) and compared to the respective native transcripts, *Pdp1* and *Slob* (black squares with black lines). Each time point represents the mean data from four independent experiments (±SEM). Each profile exhibited significant regulation (Kruskal–Wallis [*P*-value;peak]: *Pdp1*[0.007;CT12] *Pdp1 4xCATAC-luc*(0.028;CT6) *Slob*[0.023;CT6] *Slob 4xCATAC-luc*[0.026;CT6]). (**C**) The matching mRNA phases for *Slob 4xCATAC-luc* (A) versus *Pdp1 4xCATAC-luc* (B) are consistent with in-phase luciferase activity rhythms observed for the across-the-board line average (left; data from Figure 2A) or for the individual lines used in (A) and (B) (right; data from control flies in Figure 5, below).

The native *Pdp1* and *Slob* transcripts oscillate robustly in fly heads (25–28). While we had found that *Pdp1* and *Slob* 4xCATAC-*luc* oscillated in whole flies, it remained unclear whether the 4xCATAC reporter transcripts oscillated in the same phase as their respective native genes. To address this question, we performed a qRT-PCR time course analysis on native and reporter gene mRNA in whole fly heads. We found that both *Pdp1* and *Slob* 4xCATAC-*luc* oscillated in phase with each other, peaking at CT6 (Figure 4A and B). This is consistent with observed in-phase luminescence patterns for reporter activity of these constructs (Figure 4C). Native *Slob* transcript also peaked at CT6, while the native *Pdp1* transcript peaked slightly out of phase with *Pdp1* 4xCATAC at CT12. The different phases of CLK/CYC-regulated *Pdp1* and *Slob* transcripts may reflect subtly different transcriptional activities of their promoters or differing mRNA half-lives (28,30).

### CATAC*-luc* reporters require *cyc* and *per* for rhythmicity, but not induction

**Figure 5.**
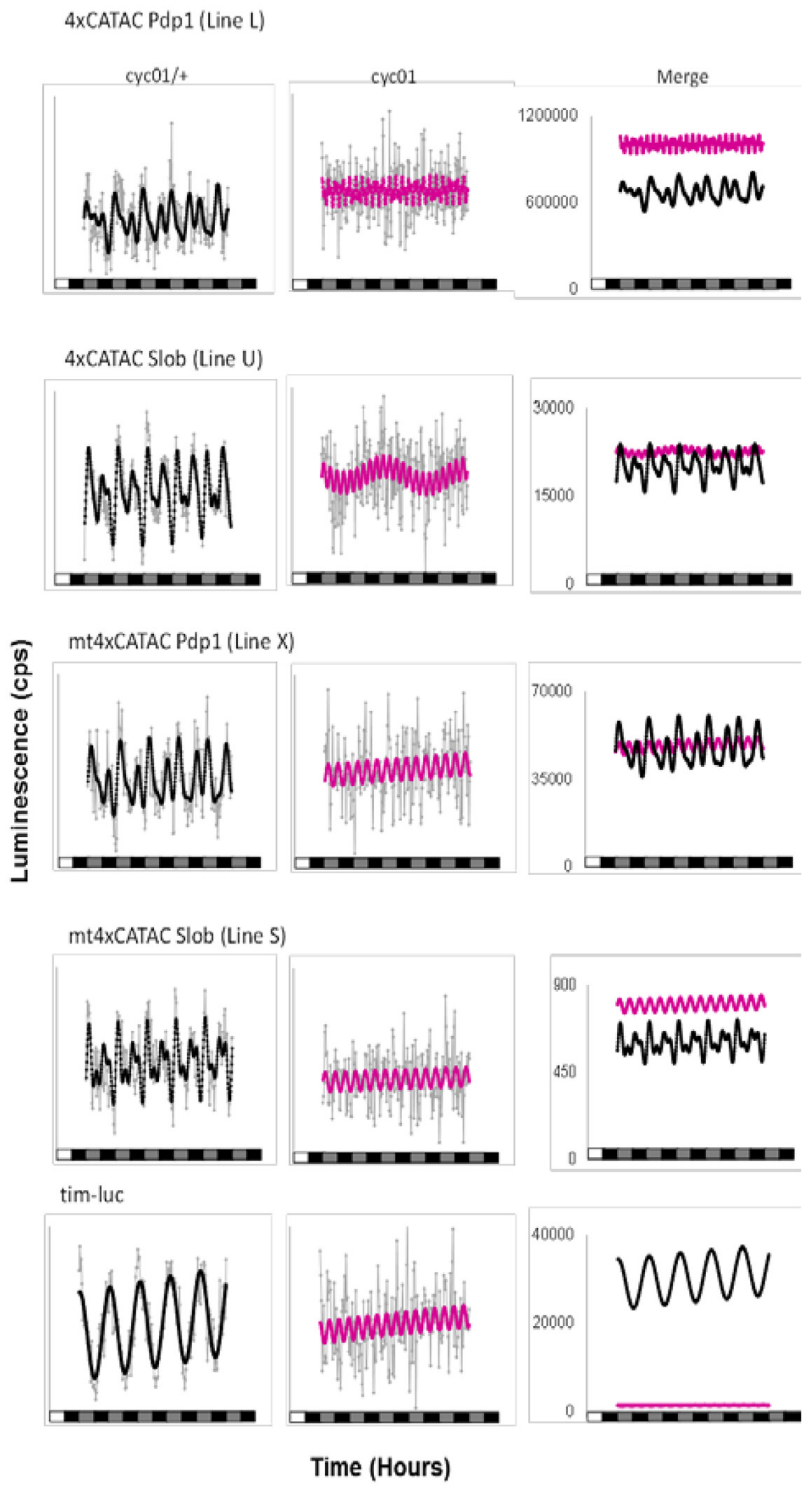
Impact of the *cyc^01^* mutation on 4xCATAC*-luc* rhythmicity and expression. Comparison of detrended, average traces (gray) and their corresponding FFT theoretical overlays (black or magenta) for a single fly line per genotype. Heterozygote controls (black) and the corresponding *cyc^01^* homozygotes (magenta) show that without a functioning clock, CATAC fails to yield circadian reporter oscillations. Residual *Pdp1* and *Slob* mt4xCATAC rhythmicity is further diminished in the homozygous *cyc^01^* genetic background. Noticeably, in the *cyc^01^* homozygote background *tim-luciferase* expression levels drop considerably, a phenomenon not observed with 4xCATAC. For statistical measures of the RAE, period and overall rhythmicity refer to Supplementary Figure S10 and Table 3.

Based on the circadian rhythmicity of 4xCATAC*-luc* reporter genes and the presence of a canonical CACGTG E-box in the CATAC consensus sequence, we hypothesized that the circadian E-box transcription factor CLK/CYC might be responsible for the observed rhythmic regulation of the CATAC element. To address this, 4xCATAC*-luc* reporter genes were introduced into a *cycle* null (*cyc^01^*) genetic background (3). As expected, *Pdp1* and *Slob* 4xCATAC lost rhythmicity in the *cyc^01^* homozygous background, as did our *tim*-luciferase positive control (Figure 5, Supplementary Figure S10, Table 3). Multimerized E-box-luc reporters also have been reported to show this in *Clk^Jrk^* flies (4). However, unlike *tim-luc*, and multi-E-box-luc constructs, *4xCATAC-luc* transgenes did not exhibit a decrease in reporter activity in *cyc^01^* homozygotes (Figure 5).

**Table 3.** Impact of the arrhythmic mutant *cyc^01^* on 4xCATAC*-luc in vivo* luminescence rhythms

Genotype	#flies	%R	%WR	%AR	Mean RAE ± SEM	Mean period ± SEM
*Pdp1 4xCATAC-luc; cyc^01^/+*	7	29	57	14	0.70 ± 0.06	25.74 ± 1.21
*Pdp1 4xCATAC-luc; cyc^01^*	14	0	14	86	0.86 ± 0.09	19.56 ± 2.59
*Slob 4xCATAC-luc; cyc^01^/+*	24	33	46	21	0.70 ± 0.04	21.98 ± 0.64
*Slob 4xCATAC-luc; cyc^01^*	23	4	35	61	0.80 ± 0.05^a^	22.68 ± 1.98
*Pdp1 mt4xCATAC-luc; cyc^01^/+*	22	9	18	82	0.73 ± 0.04^b^	23.72 ± 0.41
*Pdp1 mt4xCATAC-luc; cyc^01^*	18	0	6	94	(0.73)	(16.97)
*Slob mt4xCATAC-luc; cyc^01^/+*	24	17	29	54	0.76 ± 0.05	24.03 ± 0.68
*Slob mt4xCATAC-luc; cyc^01^*	22	0	18	82	0.85 ± 0.05^c^	18.55 ± 1.17
*pPTluc; cyc^01^/+*	24	8	38	54	0.81 ± 0.05^d^	23.66 ± 0.93
*pPTluc; cyc^01^*	20	10	25	65	0.79 ± 0.08	22.10 ± 2.19
*tim-luc; cyc^01^/+*	23	83	13	4	0.51 ± 0.04^a,b,c,d,e^	24.35 ± 0.46
*tim-luc; cyc^01^*	23	4	22	74	0.82 ± 0.05^e^	19.59 ± 1.75

ANOVA with post-hoc Tamhane's T2 *P* < 0.01^a,d^; *P* < 0.05^b,c,e^.

To better understand this result, we performed further analysis of the association between CATAC and observed CLK/CYC binding using published CLK antibody ChIP-chip data (38). CATAC elements in the core clock genes, *Pdp1, tim, per* and *cwo* as well as the clock-controlled gene, *Slob*, tended to coincide with CLK or PER ChIP signals only in the presence of either an internal canonical E-box or closely-linked external E1E2 motif (Supplementary Figure S11A and B). Moreover, an extended analysis across the entire genome exposed significant enrichment of CLK binding at E1E2 sites, but not CATAC sites (Supplementary Figure S11C; Supplementary Table S2). Taken together, although CATAC-mediated circadian rhythms depended on CLK/CYC, and it is possible that CATAC and CLK/CYC-regulated elements act cooperatively in native clock gene promoters, our results indicated that CLK/CYC is not the (only) direct regulator of CATAC. This conclusion is supported by our observation that 4xCATAC-*luc* differed from multimerized E-box-luc or tim-luc in its ability to maintain high expression levels in the absence of CLK/CYC activity and the fact that *in vivo* CLK-binding near individual CATAC elements did not appear predictive of their ability to mediate circadian rhythms, but rather of their proximity to canonical E-boxes.

**Figure 6.**
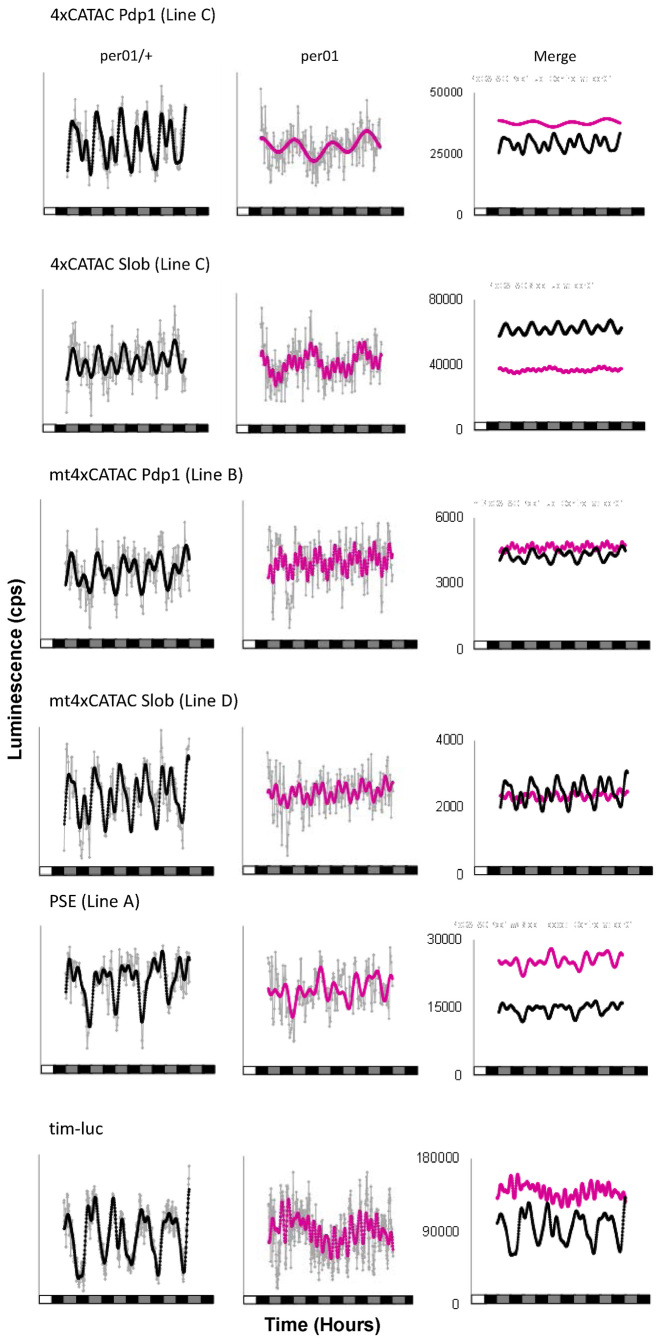
Impact of the *per^01^* mutation on 4xCATAC*-luc* rhythmicity and expression. Comparison of detrended, average traces (gray) and their corresponding FFT theoretical overlays (black or magenta) for a single fly line per genotype. PSE as well as *Pdp1* and *Slob* 4xCATAC heterozygote controls (black) and the corresponding *per^01^* homozygotes (magenta) show that without a functioning clock, CATAC fails to yield reporter oscillations. Residual *Pdp1* and *Slob* mt4xCATAC rhythmicity is further diminished in the homozygous *per^01^* genetic background. Most reporter constructs, including *tim-luc* and various CATAC*-luc* insertions, maintain similar or somewhat increased expression levels in the *per^01^* homozygous background; only *Slob* 4xCATAC expression levels exhibited a mild decrease. For statistical measures of the RAE, period and overall rhythmicity refer to Supplementary Figure S10 and Table 4.

In *Drosophila*, CLK/CYC is rhythmically repressed by the PER/TIM heterodimer to produce oscillations of clock-regulated gene transcripts such as *vri* and *Pdp1* (30). A previous study showed that in a *Clk^Jrk^* or *cyc^01^* genetic background *tim, per* and *vri* transcription is low (39)—which we confirmed with *tim* in our bioluminescence assay (Figure 5). However, 4xCATAC does not act in a similar fashion and instead remains at high to intermediate expression levels—a phenotype expected of VRI/PDP1-regulated transcripts such as *Clk* and *cry* (30). If CATAC were regulated in a fashion similar to the *Clk* and *cry* promoters, we would expect its activity to be not only arrhythmic, but also strongly reduced in a *per^01^* background, where increased VRI activity is thought to result in suppression of these promoters. In contrast, CLK/CYC-regulated promoters such as *tim* are expressed at intermediate levels in this background (27) (Figure 6). Although there was some variation among individual wild-type 4xCATAC-*luc* lines (for example, the *Slob* 4xCATAC line that was tested showed somewhat reduced expression levels in the *per^01^* background), by and large there was little effect of the *per^01^* mutation on their expression levels (Figure 6). CATAC, therefore, does not appear to be co-regulated with VRI/PDP1 elements.

Additional experiments were performed to examine whether the somewhat variable response of 4xCATAC-*luc* expression levels to the *per^01^* mutation could be explained by the presence or absence of a canonical E-box. To this aim, we tested our *Pdp1* 4xCATAC with *Slob*-like E-boxes (PSE) construct. PSE is a modified version of *Pdp1* 4xCATAC, which has E-box sequences that were mutated such that their deviation from the CACGTG consensus matched that of the four E-boxes in the *Slob* 4xCATAC enhancer (Figure 1A). PSE still showed high expression levels in *per^01^* flies, which suggests that the E-box content is not responsible for the observed differences. Nevertheless, in all cases 4xCATAC lost rhythmicity in the *per^01^* homozygotes, confirming that PER was required for CATAC-mediated rhythms (Figure 6, Supplementary Figure S12, Table 4).

**Table 4.** Impact of the arrhythmic mutant *per^01^* on 4xCATAC*-luc in vivo* luminescence rhythms

Genotype	#flies	%R	%WR	%AR	Mean RAE ± SEM	Mean period ± SEM
*Pdp1 4xCATAC-luc; per^01^/+*	24	71	25	4	0.62 ± 0.03^a^	24.92 ± 0.65
*Pdp1 4xCATAC-luc; per^01^*	23	17	13	70	0.73 ± 0.05	23.89 ± 3.00
*Slob 4xCATAC-luc; per^01^/+*	20	45	45	10	0.66 ± 0.03^b^	24.19 ± 1.25
*Slob 4xCATAC-luc; per^01^*	22	9	18	73	0.76 ± 0.05	25.18 ± 2.47
*Pdp1* with *Slob*-like E-boxes *4xCATAC-luc; per^01^/+*	21	29	48	24	0.69 ± 0.04^c^	25.37 ± 0.77
*Pdp1* with *Slob*-like E-boxes *4xCATAC-luc; per^01^*	21	5	5	90	0.63 ± 0.17	21.92 ± 4.19
*Pdp1 mt4xCATAC-luc; per^01^/+*	20	30	30	40	0.69 ± 0.04^d^	24.44 ± 1.03
*Pdp1 mt4xCATAC-luc; per^01^*	25	8	20	72	0.80 ± 0.05^e^	22.91 ± 2.63
*Slob mt4xCATAC-luc; per^01^/+*	24	63	17	21	0.59 ± 0.03^f^	23.73 ± 0.75
*Slob mt4xCATAC-luc; per^01^*	24	0	17	83	0.89 ± 0.03^a,f,g,h^	22.53 ± 2.67
*pPTluc; per^01^/+*	21	62	24	14	0.60 ± 0.04^h^	26.75 ± 0.85
*pPTluc; per^01^*	21	0	19	81	0.85 ± 0.05^i^	20.27 ± 1.51
*tim-luc; per^01^/+*	24	88	13	0	0.46 ± 0.04^b,c,d,e,g,i^	23.87 ± 0.44
*tim-luc; per^01^*	24	17	4	79	0.65 ± 0.05	18.62 ± 2.58

ANOVA with post-hoc Tamhane's T2 *P* < 0.001^g^; *P* < 0.01^e^; *P* < 0.05^a,b,c,d,f,h,i^.

## DISCUSSION

Regulation of circadian gene expression relies on a number of transcriptional elements found in the promoters and introns of core clock and clock-regulated genes. We now know of at least 5 reported functional transcriptional elements that are represented in circadianly regulated genes in *Drosophila*—the E-box, PERR element, VRI/PDP1-box, CRE element and TER box (27). Among them, the E-box is the best studied. The E-box is a rather versatile regulatory element, capable of functioning with deviations from its canonical form and utilizing flanking sequences and/or nearby E-boxes and regulatory elements to modulate its functionality (7,8).

Noncanonical E-box regulation of circadian genes has previously been identified in *Drosophila tim* and mammalian *dbp* (6,40).The CATAC element possesses an E-box sequence which may be either canonical or noncanonical in nature. Regardless, CATAC is able to generate robust, sustainable rhythms as observed with the *Pdp1* and *Slob* 4xCATAC, as well as the *Pdp1* with *Slob*-like E-boxes (PSE), constructs. Thus, the considerable variation in quality of E-box sequences between these constructs did not obviously impact reporter gene rhythms. The sequence immediately flanking an E-box element has also been implicated in affecting regulation (9). Simultaneous mutation of residues in the conserved core region of CATAC as well as its E-box, resulted in disrupted rhythms and decreased reporter expression. This suggests that the conserved core sequences are important to CATAC regulation, although it is unclear as to whether this corresponds to rhythmicity, expression levels or both. This question may be addressed in future studies by mutational analyses of only the CATAC core sequence.

As previously observed by McDonald *et al.* (6), transcriptional elements nearby an E-box can influence circadian gene regulation. The *tim* promoter has a canonical E-box that relies on two proximal cis-regulatory elements that can also interact with CLK/CYC to produce rhythmic transcriptional activity. Genomic analysis of CATAC showed that a high likelihood of CLK/CYC binding coincided with the presence of either a canonical E-box internal to CATAC or the presence of E1E2 motifs in close proximity (Supplementary Figure S11).

For all the similarities that CATAC shares with E-box motifs, CATAC is not simply another E-box element. Normally, an E-box regulated transcriptional element exhibits low reporter expression in a *cyc^01^* genetic background and intermediate-to-high expression in *per^01^* (3,41). CATAC shows high reporter expression in a *cyc^01^* background and generally intermediate-to-high expression in *per^01^*. While CATAC rhythmicity relies on a functioning core clock, CLK/CYC does not appear to be the only potential regulator of CATAC. There is likely another regulator, or regulators, that act on the CATAC to generate increased transcriptional activity in the null backgrounds. Other E-box-binding transcription factors may contribute to the regulation of CATAC along with transcription complexes binding to the conserved core sequence. In particular, the basic helix-loop-helix transcription factor CLOCKWORK ORANGE (CWO) is known to bind E-boxes in clock-controlled genes (42,43) and may, therefore, modulate CATAC activity. However, since CWO exhibits an inhibitory effect on E-box-mediated transcription it is unlikely to be directly responsible for the enhancer activity of CATAC elements.

Thus, we have discovered a novel circadian regulatory element that, although it possesses an E-box-like motif, exhibits non-E-box-like responses. Through use of multimerized CATAC element, we concluded that CATAC is capable of contributing to the rhythmicity of clock genes, such as *Pdp1* and *Slob*. Of course, this conclusion comes with the caveat that the multimerized constructs do not represent the complexity of the native genomic environment. Mutational analysis of CATAC sequences in their natural context will constitute an important next step in the functional analysis of this *cis*-regulatory element. Nevertheless, the identification of CATAC contributes to our knowledge of circadian transcriptional elements, and may be used to further characterize the regulatory regions of clock and clock-regulated genes.

## Supplementary Material

Supplementary Data
