# Supplementary material for: A new promoter element associated with daily time keeping in Drosophila
Source: Nucleic Acids Res. 2017 Apr 12;45(11):6459–70. doi: 10.1093/nar/gkx268 (PMC5499816; doi:10.1093/nar/gkx268)
Supplement: Supplementary Data [file gkx268_Supp.zip › SharpPaquetNaefBafnaWijnen120417supplement.pdf]

Supplementary Materials

FIGURE S1

a)

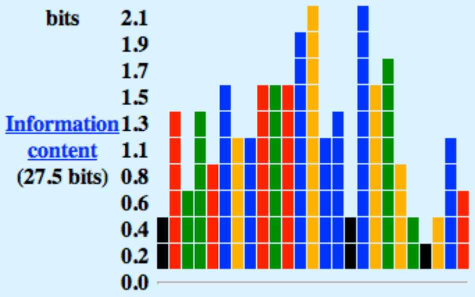

Multilevel consensus sequence

CATTACGCATACGCCACGTGTAGCA
A G T G TGT
G

NAME	STRAND	START	P-VALUE		SITES	
ana_pdp1	+	415	7.79e-15	TGGACGAGCA	CATTACGCATACGTCACGTGTTGCA	GACGCATCAG
mel_pdp1	+	387	7.79e-15	CTTTGGAGCA	CATTACGCATACGTCACGTGTTGCA	GACGCACCAG
gri_pdp1	+	554	7.77e-14	TTTTCCATGA	AATTACGCATACGTCACGTGTTGCA	GGCCAATTAA
moj_pdp1	+	450	7.77e-14	CTTTCCATGA	AATTACGCATACGTCACGTGTTGCA	AGCCAATTAA
vir_pdp1	+	489	1.61e-13	ATTTCCATTA	AATTGCGCATACGTCACGTGTTGCA	AGCCAATTAA
pse_pdp1	+	319	3.02e-12	AGTCGCATCA	CATTGCGCCTACGTCACGTGTTGCA	CACGCATCAA
vir_stich1	-	10	7.61e-12	CGGTTCTTGG	CGTTACGCATACGCCACGTGTACAA	TTCAAAGAA
ana_tim	+	157	4.03e-11	GCAGGAAGTG	CGATGCGTATACGCCGCGTGTGGCA	GCAACCAACT
gri_tim	+	151	5.63e-11	TTTGTGTATG	CATTGCGCATACGCCGCGTTAATCG	AGCAATTGTT
pse_tim	-	88	6.99e-11	GTAACGCGAA	AAATGCGCATACGCCACATGTATCA	CACCTGAATC
mel_per	+	1679	1.61e-10	TCTGGCCGCA	GAATACGCATACGCAACGTGGTGCA	CCACTGCAGC
mel_tim	-	88	1.78e-10	TAATGCAAGT	AATTGCGTATACGCCGCATGTAACA	CATGAACGGT
moj_stich1	-	221	2.40e-10	CCTATTCACA	CATTACACATACGCCCCGTAAGGCA	CGTGCGCGTX
ana_stich1	-	234	3.53e-10	TTCGAGCGGA	TATTACACATACGCCCCGTGGGCCC	TGTTGAGGGX
vir_tim	+	18	6.78e-10	TACATTTGCA	CATTGCGCATACGCCGCGTTAATTG	ACCAACTGTT
moj_tim	+	91	3.73e-09	CATTGCATTT	CATTGCGCATACGCCTCGTTAGCTG	CCCAACTGTT
ana_per	+	1313	8.72e-09	ACTCGCTCAA	TATTACGCATACGTAGCGTTGCACC	AGATGGTTNN
ana_vri	-	178	2.55e-08	XCGTGTTTTG	CAGAACGCACGCGTCACATGTAGAA	ACGTCTGCCG
gri_stich1	-	221	5.65e-08	TAAACCCGGT	TATTACAAATACGCTGCGATGGGCA	CGTGTGCATX
pse_stich1	-	479	1.12e-07	ATAATCAAAA	TAATAATCATACGCTGCGTTGTCCC	GTCATCGATG
mel_vri	-	174	1.19e-07	XTGTATTTTG	CAGAACTCACGCGTCACATGTAGAA	ATGTCTGCGG
mel_stich1	-	217	1.91e-07	CCTTGTACGG	CGTTATGTATACGCCCCGTTCGGAT	GTTACACATA
moj_per	+	351	3.10e-05	AACCTCAATN	NNNNNNNNNTATGCCACGTGTAACC	CGGAAAAGTC

b)

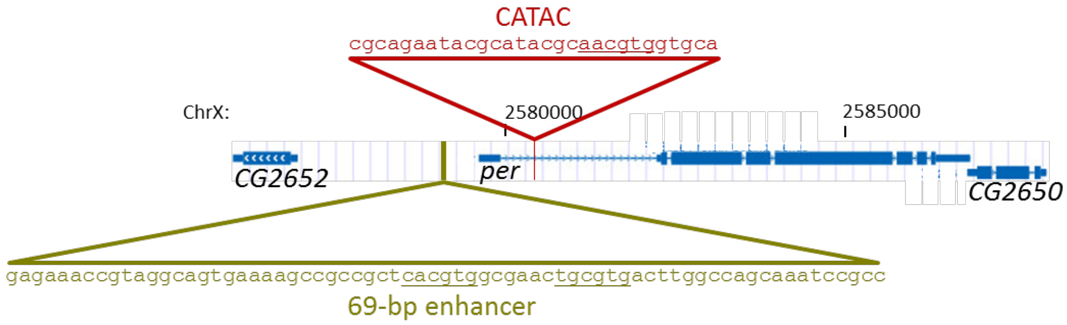

Individual CATAC elements and consensus. a) Alignment of CATAC elements from the *Pdp1*, *tim*, *per*, *cwo* (annotated as *'stich1'*) and *vri* genes of 12 species of *Drosophila.* Note that the first 4 nucleotides of the 29-bp CATAC element are not color-coded here. b) Genomic location of the CATAC element and the previously described 69-bp enhancer in the *D. melanogaster per* gene.

FIGURE S2

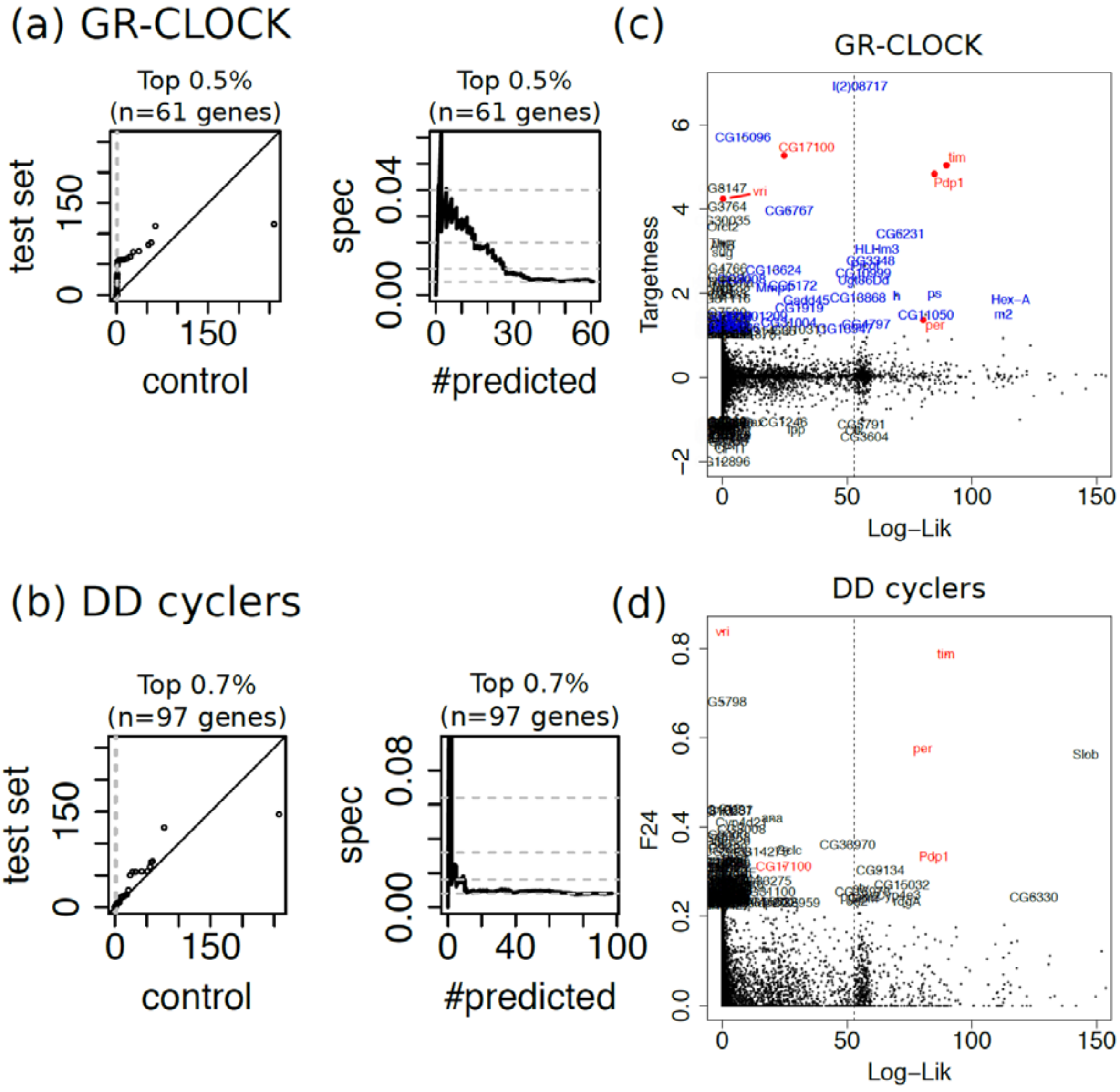
(a) GR-CLOCK
Top 0.5%
(n=61 genes)
test set
control
spec
#predicted
(b) DD cyclers
Top 0.7%
(n=97 genes)
(c)
GR-CLOCK
Targetness
Log–Lik
tim
Pdp1
CG17100
CG16096
vri
CG6767
CG6231
HLHm3
Hex–A
m2
ps
per
CG11050
(d)
DD cyclers
F24
Slob
CG6330
CG38970
CG9134
CG15032

(e)

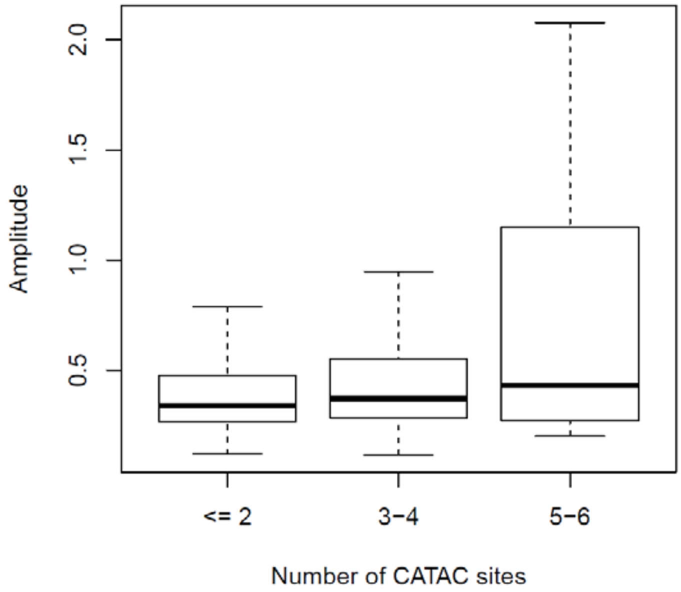

Genes with strong induction by a GLUCOCORTICOID RECEPTOR-CLOCK (GR-CLK) fusion or strong circadian rhythmicity in DD are enriched for CATAC elements. The Log-Likelihood (Log-Lik) of the CATAC element model was used to estimate CATAC overrepresentation ±2,500 bp around the Transcription Start Site (TSS) of individual genes. **(a-b)** Left-hand diagrams: quantile–quantile plots showing overrepresentation of CATAC elements, as measured by an upshift in Log-Lik scores in highly induced (a) or cycling (b) genes (positives) with respect to the control (negatives). Positives correspond to the 61 induced (a) (upper left diagram) or 97 cycling (b) (lower left diagram) genes while the negative set consists of all remaining genes. Right-hand diagrams: Specificity versus number of predicted genes (sensitivity) in the group of 61 induced (a) (upper right diagram) or 97 cycling (b) (lower right diagram) genes. The grey dashed horizontal lines represent expected specificity (lowest line), 2-fold, 4-fold, and 8-fold enrichment. Importantly, the five training genes are excluded from the set of positives in all panels. **(c-d)** Scatter plot representing the targetness score (fold induction in $\log_2$ units by GR-CLK) or 24h Fourier component (for DD rhythms), respectively, in function of Log-Lik scores. Genes in blue in (c) are the top 30 genes (from the group of 61 induced genes) with highest match to the sequence model. Genes in red are the 5 training genes. A file with the data for panels (c) and (d) is included as a separate supplement. **(e)** Genes with more CATAC sites have larger daily amplitude. Boxplots displaying gene amplitude distribution (mean range of normalised LD and DD rhythms) in function of the number of CATAC sites (bit score >= 10) around TSS (±2500 bp). Significance was assessed using a Kruskal-Wallis non-parametric test (P = 0.029). Note that the more relaxed bit score threshold allowed partial CATAC matches to be taken into account. Elsewhere in this paper bit score thresholds of 20 for matches and 30 for high quality matches were used.

FIGURE S3

Slob 4xCATAC enhancer

5′pCCGATAACGCGGCGTATGCGCAATGTCGAAGCATATTACGCATACGCCCCATCCGCCACAGCAAACGCTGCGTATGCGTAATACTTTGTGCACACGTTGCGTATGAGTAATGTCCT3′

Slob mt4xCATAC enhancer

5′pCCGATATCCCGGCCTTTGCCCTATGTCGAAGCATATAAGGCAAAGGCCCGAACCGCCACAGCAATCCCTGCCTTTGCCTTATACTTTGTGCACTCCTTGCCTTTGACTTATGTCCT3′

Pdp1 4xCATAC enhancer

5′pAGCACATTACGCATACGTCACGTGTTGCAAAAATAATACGTATACGACGCGTGTCTTCTGTACCATGCGGCGTATGAGCAATCTGTTAATACGTTACCCATACGCCCCGTGGGCC3′

Pdp1 mt4xCATAC enhancer

5′pAGCACATAAGGCAAAGGTCAGGAGTTGCAAAAATAAAAGGTAAAGGACGGGAGTCTTCTGTACCTTCCGGCCTTTGACCTATCTGTTAATACGTAAGCCAAAGGCCCGGAGGGCC3′

Pdp1 4xCATAC with Slob-like E-boxes enhancer

5′pAGCACATTACGCATACGTAACGTGTTGCAAAAATAATACGTATACGAAGCGTTTCTTCTGTACGATGGGGCGTATGAGCAATCTGTTAATACGTTACCCATACGCCGCGTTGGCC3′

Sequences for the assembled 4xCATAC enhancer inserts (excluding flanking restriction sites). Blue and red typeface indicate deviations from the consensus due to natural variation and mutation, respectively.

FIGURE S4

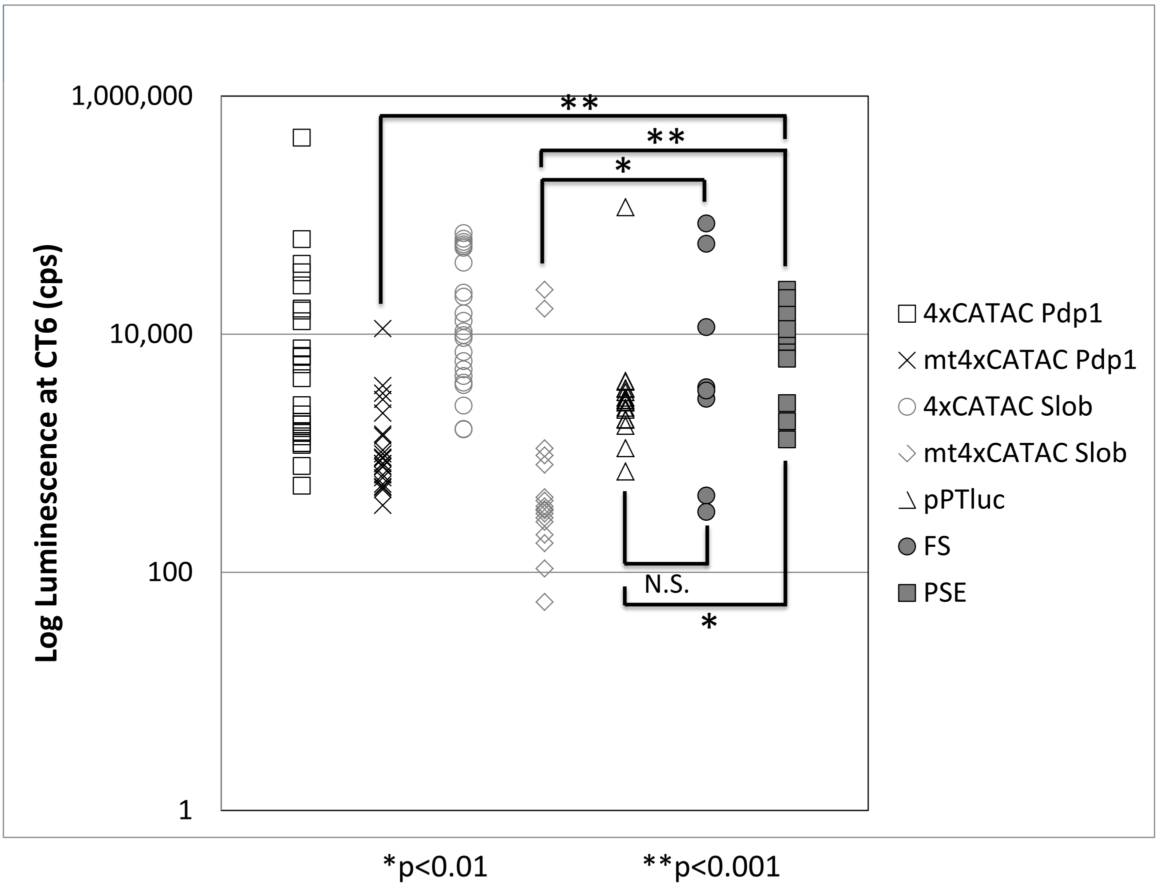

Mann-Whitney U/Wilcoxon W rank sum test. Expression level values are higher for PSE than *Pdp1* mt4xCATAC, *Slob* mt4xCATAC and pPT*luc*. Remaining comparisons of PSE to *Pdp1* 4xCATAC (p=0.09), *Slob* 4xCATAC (p=0.40), and FS (p=0.44) are not significant. Expression level values are higher for FS than *Slob* mt4xCATAC. Remaining comparisons of FS to *Pdp1* 4xCATAC (p=0.93), *Pdp1* mt4xCATAC (p=0.07), *Slob* 4xCATAC (p=0.21) and pPT*luc* (p=0.29) are not significant.

FIGURE S5

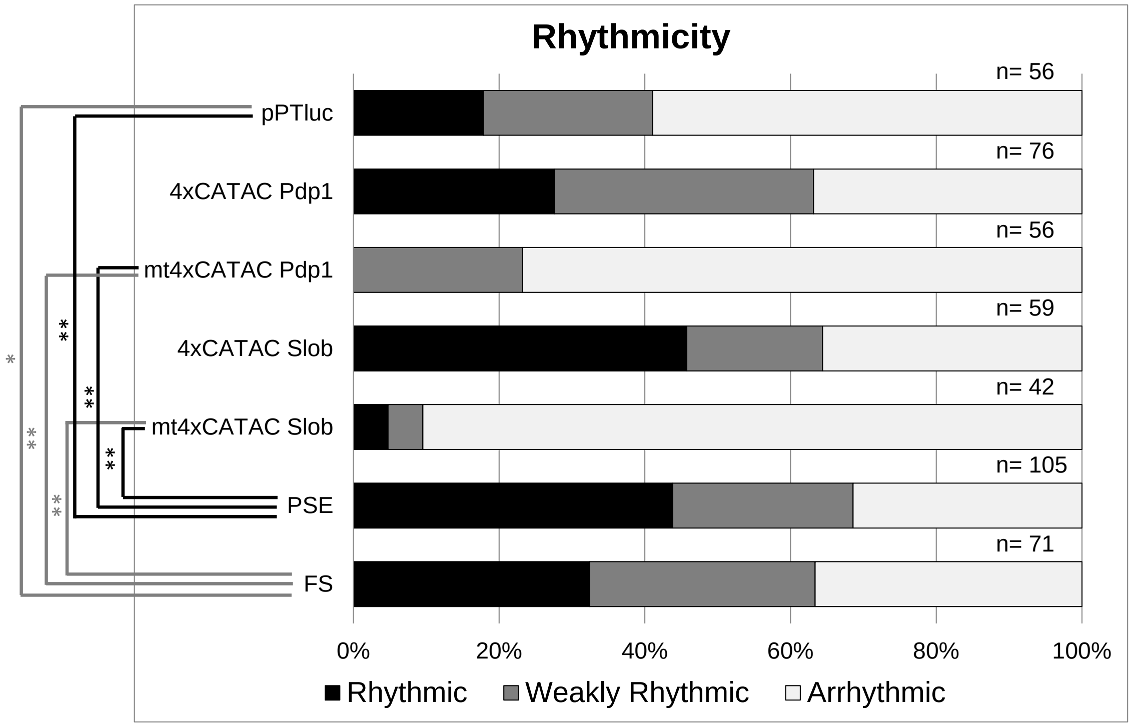

PSE = Pdp1 4xCATAC with Slob-like E-boxes; FS = Flipped Slob 4xCATAC

*p<0.05 **p<0.001

Pairwise chi-square tests for overall rhythmicity. FS and PSE generate greater rhythmicity than *Pdp1* mt4xCATAC, *Slob* mt4xCATAC and pPT*luc*. FS is not significantly different from either *Pdp1* 4xCATAC (p=0.78) or *Slob* 4xCATAC (p=0.18). PSE is not significantly different from either *Pdp1* 4xCATAC (p=0.07) or *Slob* 4xCATAC (p=0.65). PSE and FS are also not significantly different from one another (p=0.31).

FIGURE S6

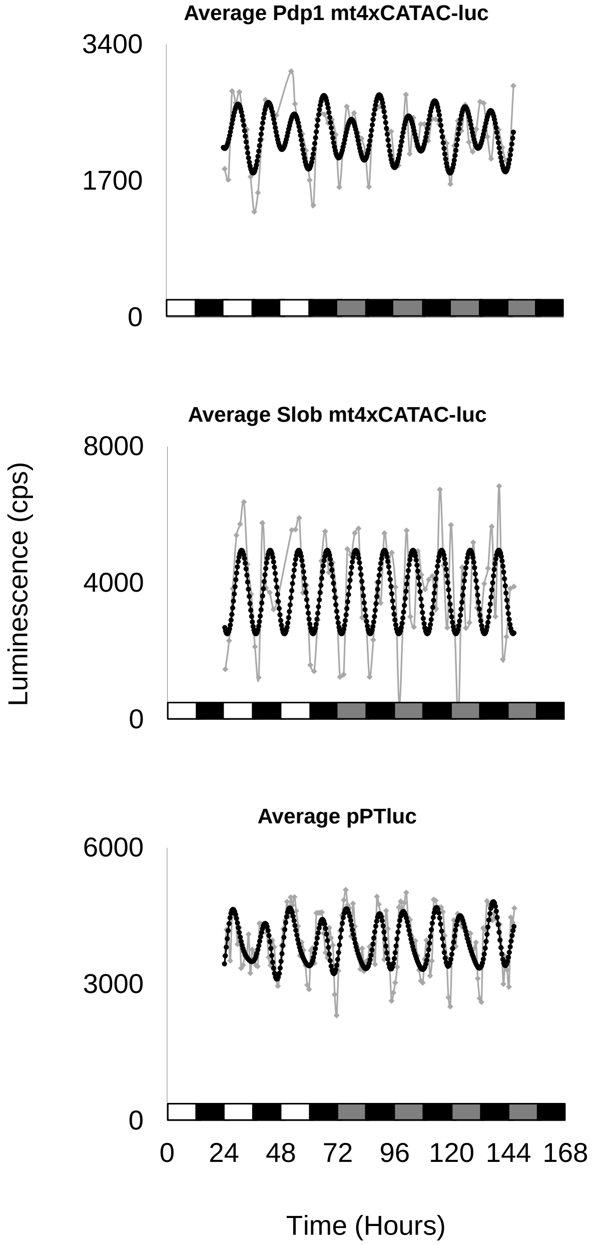

Comparison of detrended, average traces (gray) and their corresponding FFT theoretical overlays (black) demonstrate low level expression and 12-hr rhythms in flies with *Pdp1* and *Slob* mt4xCATAC. Empty vector, pPT*luc*, shows similar expression levels and activity.

FIGURE S7

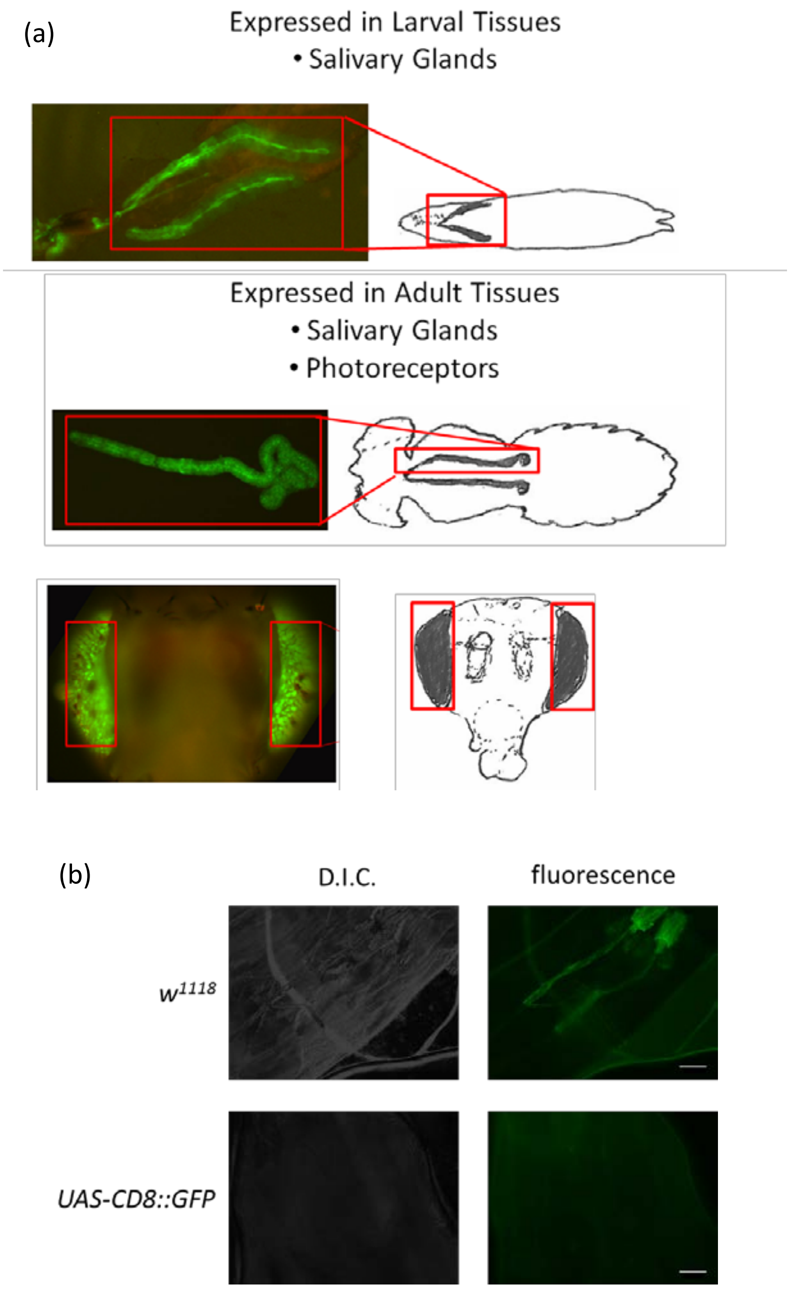

(a) *Pdp1* and *Slob 4xCATAC-Gal4* expression. Larvae and adult flies expressing *Pdp1* and *Slob 4xCATAC-Gal4* driven *UAS-CD8::GFP* show expression primarily in the salivary glands in addition to the photoreceptor cells of the compound eye in the adult. Mutant *Pdp1* and *Slob* 4xCATAC-*Gal4* lines also share this expression pattern as demonstrated in Figure 3a. **(b)** Observed larval salivary gland fluorescence was specific to transgenic induction by *Pdp1* and *Slob* 4xCATAC-*Gal4*. To verify that the larval salivary gland signals described in panel (a) were not due to autofluorescence or leaky expression of the *UAS-CD8::GFP* construct, third-instar larvae from non-transgenic (w^{1118}) or *Gal4*-negative (*UAS-CD8::GFP*) controls were imaged for fluorescence of their salivary gland tissue. Although some relatively weak autofluorescence of tracheal tissue is visible in the fluorescence image for w^{1118} (top right panel), no specific fluorescence was observed in the salivary glands for these two control genotypes.

FIGURE S8

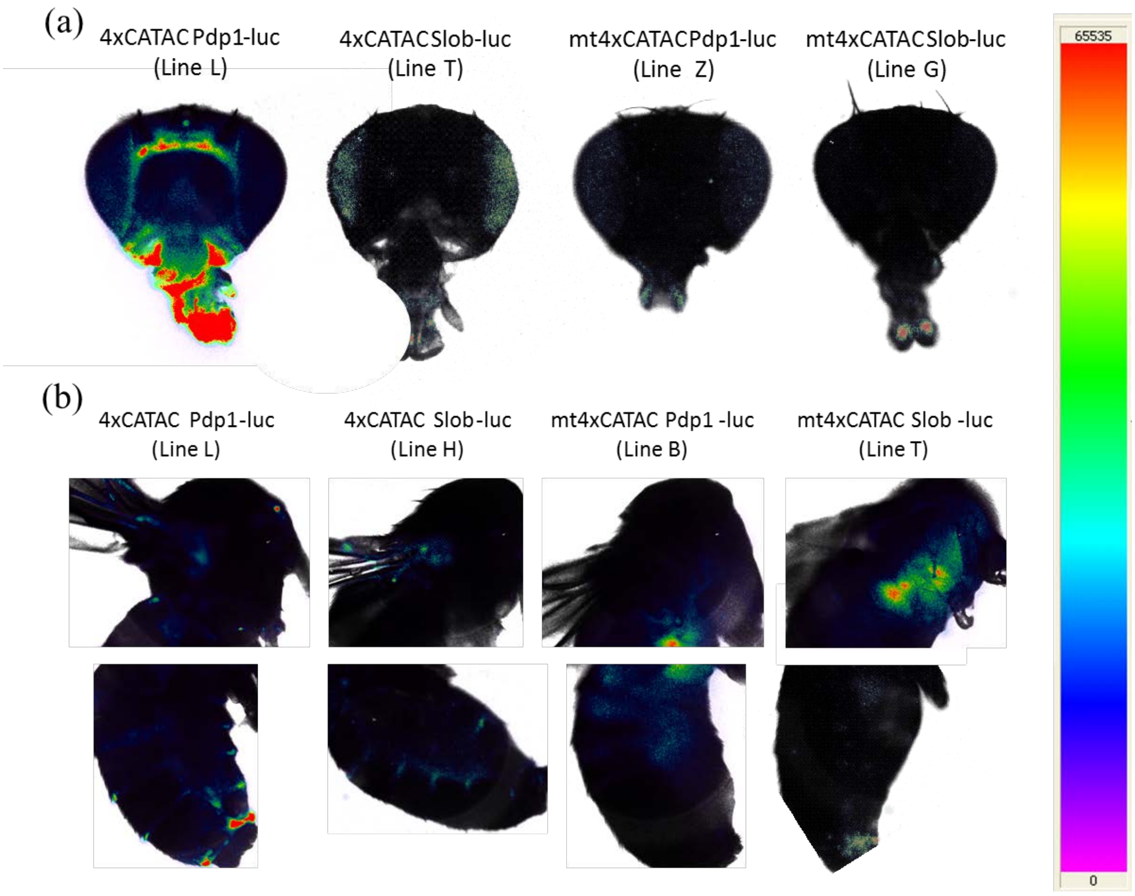

Pdp1 and *Slob* 4xCATAC-*luc* luminescence. Sample images are shown for individual fly lines. (a) Fly head (b) Fly bodies. Signal intensity scale indicted on the right.

FIGURE S9

(a)

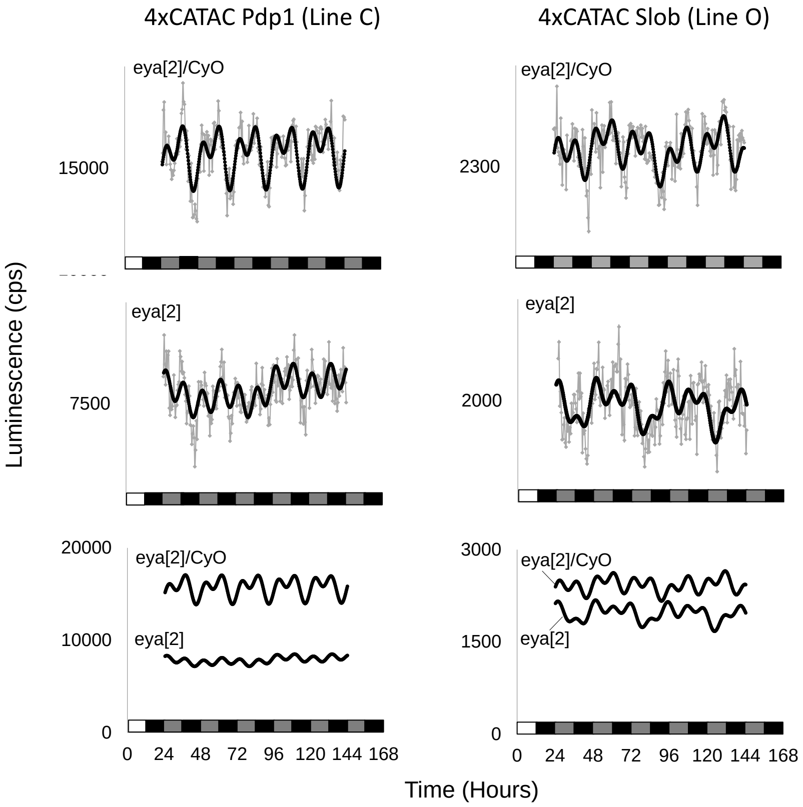

4xCATAC Pdp1 (Line C)
4xCATAC Slob (Line O)
eya[2]/CyO
15000
eya[2]/CyO
2300
Luminescence (cps)
eya[2]
7500
eya[2]
2000
20000
eya[2]/CyO
10000
eya[2]
0
3000
eya[2]/CyO
1500
eya[2]
0
0 24 48 72 96 120 144 168
0 24 48 72 96 120 144 168
Time (Hours)

(b)

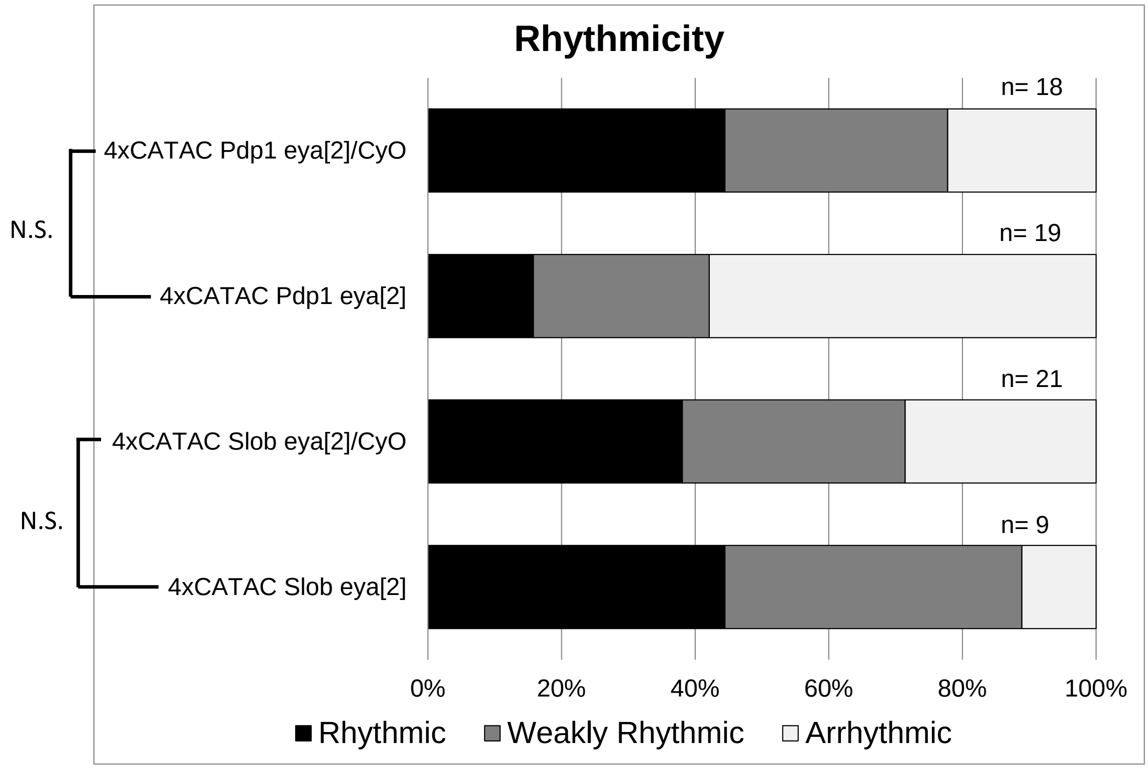

Rhythmicity
n= 18
4xCATAC Pdp1 eya[2]/CyO
N.S.
n= 19
4xCATAC Pdp1 eya[2]
n= 21
4xCATAC Slob eya[2]/CyO
N.S.
n= 9
4xCATAC Slob eya[2]
0% 20% 40% 60% 80% 100%
Rhythmic Weakly Rhythmic Arrhythmic

Comparison of 4xCATAC-*luc* reporter expression in *eya*^{2} mutant and control flies. (a) Detrended, average traces (gray) and their corresponding FFT theoretical overlays (black) for the indicated fly lines. *Pdp1* and *Slob* 4xCATAC *eya*^{2} heterozygote controls and the corresponding *eya*^{2} homozygotes show that without eyes, 4xCATAC flies produce less luciferase signal at CT6 (Mann-Whitney U/Wilcoxon W rank sum test, *Pdp1* 4xCATAC $p<0.001$, *Slob* 4xCATAC $p=0.05$). Between manipulations, the RAE, period and overall rhythmicity are not statistically different (refer to Table 2). (b) Pairwise chi-square tests for overall rhythmicity. *Pdp1* 4xCATAC (Line C) in the absence (*eya*^{2}*/CyO*) and presence (*eya*^{2}) of the eyes absent mutation does not show a statistically significant change in rhythmicity ($p=0.06$). *Slob* 4xCATAC (Line O) in the absence and presence of the eyes absent mutation does not show a statistically significant change either ($p=0.58$).

FIGURE S10

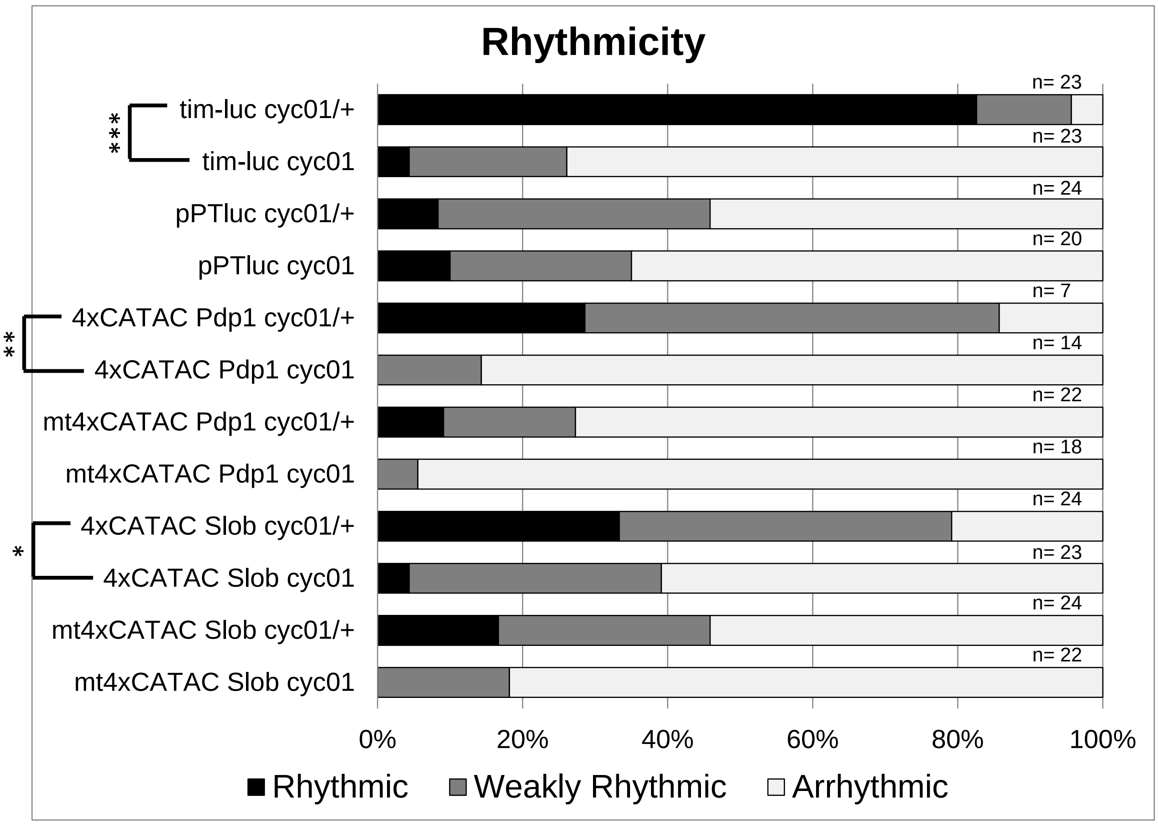

*p<0.01 **p<0.005 ***p<0.001

Pairwise chi-square test for overall rhythmicity. The *tim-luc*, *Pdp1* (Line L) and *Slob* (Line U) 4xCATAC *cyc*^{01} heterozygotes generate greater rhythmicity than their *cyc*^{01} homozygote counterparts. Differences in the residual rhythmicity of *Pdp1* (Line X) and *Slob* (Line S) mt4xCATAC *cyc*^{01} heterozygotes when compared to *cyc*^{01} homozygotes are not statistically significant (p=0.3, p=0.08; respectively). Residual rhythms persist in pPT*luc* (Line 3) empty vector even in the homozygous cyc^{01} genetic background; however, the differences in rhythmicity are not statistically significant (p=0.7). Empty pPT*luc* vector appears to generate an insignificant rhythmic component.

FIGURE S11

(a)

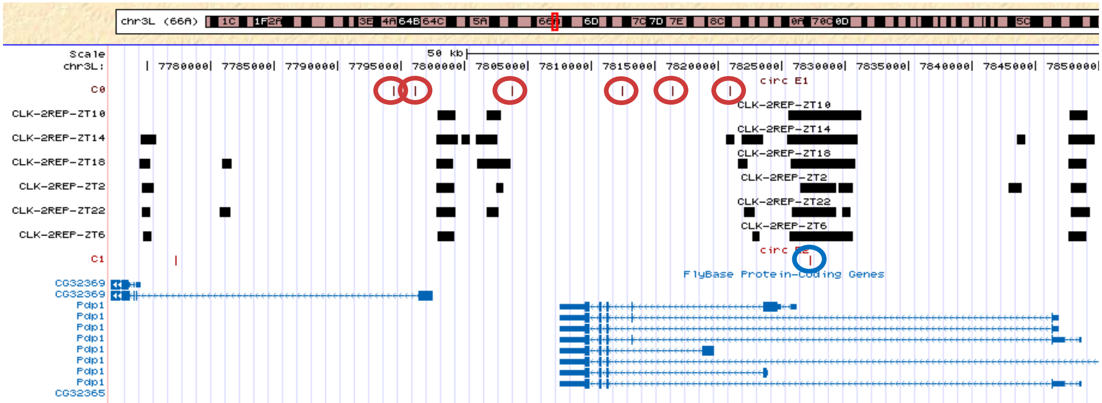

(b)

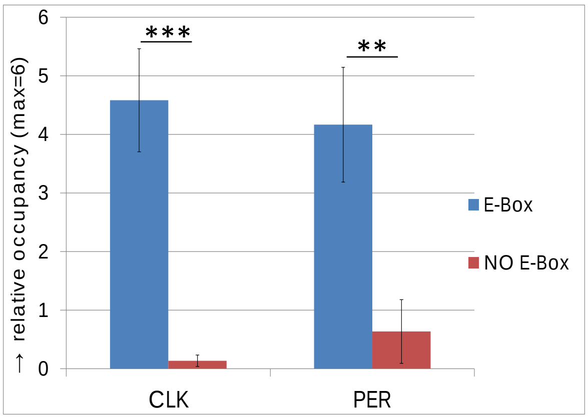

(c)

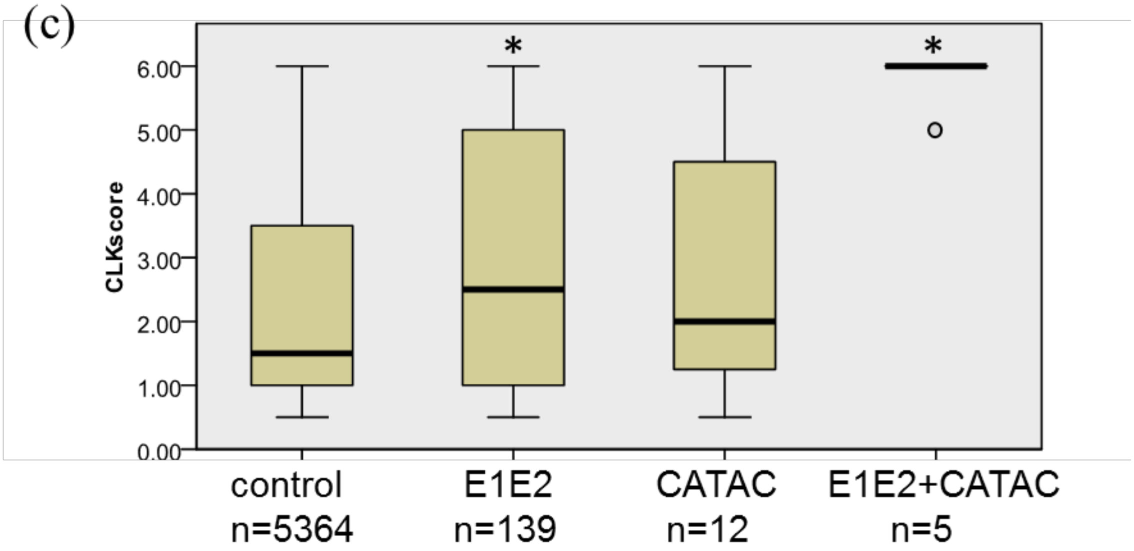

CLK/CYC and PER binding to CATAC elements depends on the presence of a consensus E-box. Hits (bit score >=20) to the CATAC consensus sequence in the proximity of the core clock gene promoters *per, tim, Pdp1,* and *cwo* as well as the clock-controlled gene *Slob*, were examined for in vivo binding by CLK and PER as annotated in the genome-wide ChIP of Abruzzi and colleagues (Abruzzi et al., 2011). **(a)** Example for CLK-binding across 6 daily time points at the *Pdp1* locus annotated with information from the UCSC genome browser as well as custom tracks for CATAC sites (C0: lacking a consensus E-box; C1: including a CACGTG E-box) and antiCLK-V5 ChIP at time points ZT2,6,10,14,18,22 (horizontal black bars). **(b)** Comparison of the relative occupancy (average ± SEM; across 0-6 of the daily time points) of chromatin surrounding CATAC sites in the presence or absence of a consensus E-box. The data was collected for clock gene promoters known to be directly regulated by CLK/CYC (*Pdp1, tim, per, cwo*) as well as the clock-controlled gene, *Slob*, which contains 4 functional CATAC sites. Note that there is little evidence of binding of CLK or PER near CATAC sites in the absence of CACGTG E-boxes. Relative occupancy of CATAC-containing chromatin by CLK and PER is significantly increased in the presence of a consensus E-box (Mann-Whitney rank-sum test: *** [$p<0.001$], ** [$p<0.01$]). **(c)** Association of CLK ChIP signal with the presence of E1E2 and high quality CATAC enhancer element matches (bit score >=30). When collapsed across the different time points, the CLK ChIP data set encompassed 5520 contiguous non-overlapping CLK-bound regions – 139 of these contained a match to the CLK/CYC binding site E1E2, 12 matched the CATAC consensus site and 5 contained a match to both. CLKscore (ranging from 0.5 to 6) refers to the coverage of the CLK ChIP ‘contigs’ across the 6 time points. The Kruskal Wallis test with additional pairwise comparisons was performed to detect and effect of the presence of the E1E2 or CATAC enhancer element on CLKscore. The presence of E1E2, but not CATAC resulted in a significantly higher CLKscore.

FIGURE S12

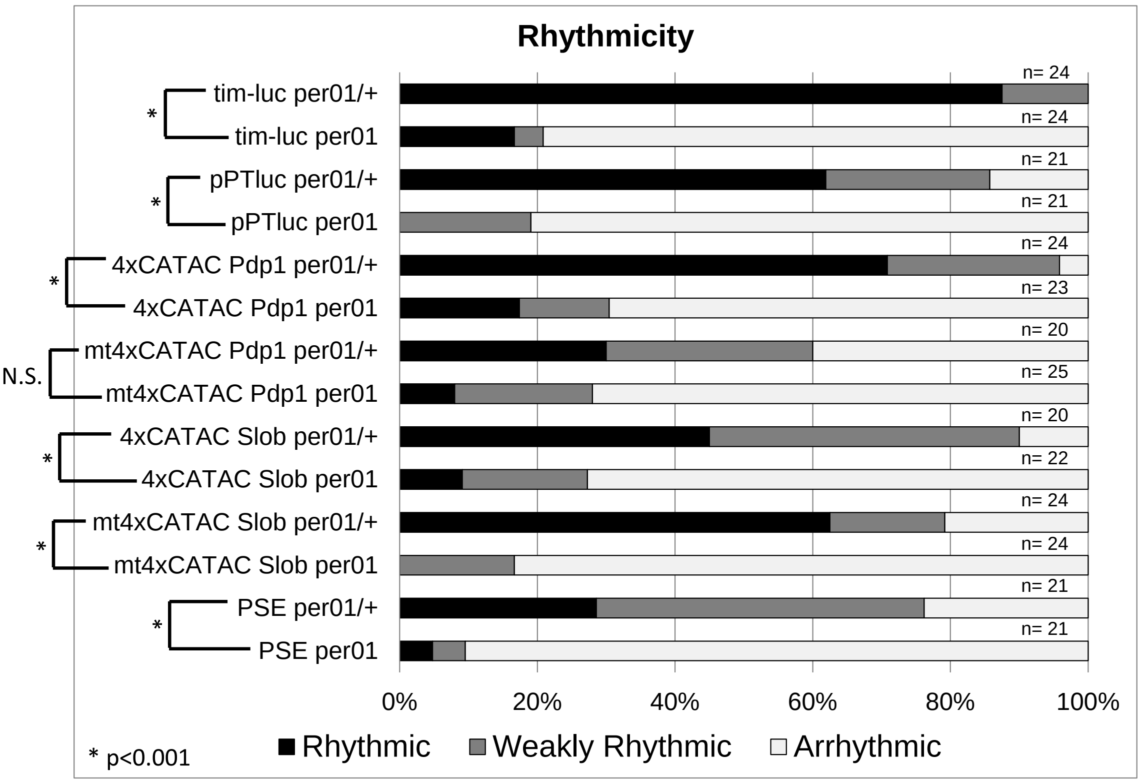

Pairwise chi-square test for overall rhythmicity. The *tim-luc*, *Pdp1*4xCATAC (Line C), *Slob* 4xCATAC (Line C) and PSE (Line A) heterozygotes generate greater rhythmicity than their per^{01} homozygote counterparts. Residual rhythmicity of *Slob* mt4xCATAC (Line D) and pPT*luc* (Line 3) is further diminished in per^{01} homozygotes. Differences in the residual rhythmicity of *Pdp1* mt4xCATAC (Line B) heterozygotes when compared to per^{01} homozygotes are not statistically significant (p=0.06)

TABLE S1

Target	Primer 1 (5’-3’)	Primer 2 (5’-3’)
Pdp1 mRNA<br>213 bp (amplicon size)<br>0.2μM (primer concentration)<br>62°C (annealing temperature)	TCTTGGCCACATAACCACAA	ATCAAAAAGTCACGCAAGCA
Slob mRNA<br>180 bp<br>2μM<br>62°C	TGCGCAACAGCTCGTATAAC	ACGACAGATTCTGGAGGCAC
luc<br>100 bp<br>1.0μM<br>60°C	TACCGGGAAAACGCTGGGCG	GGCGTTGGTCGCTTCCGGATT
Ef1-β<br>100 bp<br>0.4μM<br>60°C	GTCATCGAGGACGACAAGGT	TCTTGTTGAAGGCAGCAATG

TABLE S2

	CLK ChIP signal [-]	CLK ChIP signal [+]
E1E2 element	1635	139
CATAC element	286	12
rest of genome	88615 (estimated)	5359

Distribution of E1E2 and high quality (bit score>=30) CATAC elements across CLK ChIP-positive and -negative genome regions. Chi Square analysis (with or without Yates correction) demonstrates that CLK ChIP signal is significantly enriched at E1E2 versus CATAC ($p<0.05$) elements (5 co-localized E1E2+CATAC elements were excluded). The same test also detected enrichment of CLK ChIP signal for E1E2 regions ($p<0.001$), but not CATAC regions in comparisons to a background model for the rest of the genome (5359 CLK ChIP+ regions plus an estimated 88615 CLK ChIP- regions of similar size).
